# Mixed Model Methods for Genomic Prediction and Variance Component Estimation of Additive and Dominance Effects Using SNP Markers

**DOI:** 10.1371/journal.pone.0087666

**Published:** 2014-01-30

**Authors:** Yang Da, Chunkao Wang, Shengwen Wang, Guo Hu

**Affiliations:** Department of Animal Science, University of Minnesota, Saint Paul, Minnesota, United States of America; Harvard Medical School, United States of America

## Abstract

We established a genomic model of quantitative trait with genomic additive and dominance relationships that parallels the traditional quantitative genetics model, which partitions a genotypic value as breeding value plus dominance deviation and calculates additive and dominance relationships using pedigree information. Based on this genomic model, two sets of computationally complementary but mathematically identical mixed model methods were developed for genomic best linear unbiased prediction (GBLUP) and genomic restricted maximum likelihood estimation (GREML) of additive and dominance effects using SNP markers. These two sets are referred to as the CE and QM sets, where the CE set was designed for large numbers of markers and the QM set was designed for large numbers of individuals. GBLUP and associated accuracy formulations for individuals in training and validation data sets were derived for breeding values, dominance deviations and genotypic values. Simulation study showed that GREML and GBLUP generally were able to capture small additive and dominance effects that each accounted for 0.00005–0.0003 of the phenotypic variance and GREML was able to differentiate true additive and dominance heritability levels. GBLUP of the total genetic value as the summation of additive and dominance effects had higher prediction accuracy than either additive or dominance GBLUP, causal variants had the highest accuracy of GREML and GBLUP, and predicted accuracies were in agreement with observed accuracies. Genomic additive and dominance relationship matrices using SNP markers were consistent with theoretical expectations. The GREML and GBLUP methods can be an effective tool for assessing the type and magnitude of genetic effects affecting a phenotype and for predicting the total genetic value at the whole genome level.

## Introduction

Genomic prediction using genome-wide single nucleotide polymorphism (SNP) markers has been shown to be a powerful tool to capture small genetic effects dispersed over the genome for predicting an individual’s genetic potential of a phenotype [1]–[5]. Current large scale genomic prediction focused on additive effects [2], [4], [5]. Two SNP models for genomic prediction of additive effects were described: a traditional quantitative genetics model and a model with (−1)-0–1 SNP coding [2]. The traditional quantitative genetics model is attractive because it is equivalent to a conventional animal model with the relationship matrix calculated from the SNP genotypes [5] and it directly predicts genomic breeding values [2], [4], [5]. Method and computing tool are available for estimating genomic heritability using genome-wide SNP markers [6]. This method uses a standardization of the 0–1–2 additive coding and the subtraction step of this standardization leads to additive effects that are breeding values under the traditional quantitative genetics model assuming Hardy-Weinberg equilibrium [2], [6], [7]. The mixed model implementation of this method is ideal for a large number of markers but is not ideal for a large number of individuals because the size of the matrix that needs to be inverted increases as the number of individuals increases.

From the point of view of missing heritability [8]–[10], the ability to estimate genome-wide dominance contribution will help determine the total genetic contribution to a phenotype. Similarly, methods of genomic prediction taking into account of dominance can predict an individual’s total genetic potential for phenotypes affected by additive and dominance effects. Substantial dominance effect should justify the inclusion of dominance in genomic prediction and the design of mating systems to maximize dominance effect. In dairy cattle, dominance variances estimated from pedigree data were reported to be 11–16% of the phenotypic variance of stature [11], and the increased availability of cows with phenotypes and genotypes provides an opportunity to estimate dominance effects and include those in mating programs [12]. However, only limited methodology studies on genomic prediction and variance component estimation of dominance were available [13]–[16].

Genomic best linear unbiased prediction (GBLUP) and various Bayesian methods are available for genomic prediction, and GBLUP generally had good performance in real data [17]. Restricted maximum likelihood estimation (REML) [18] has been a widely accepted method for estimating variance components.

Objectives of this study were to develop mixed model methods for the joint genomic prediction of and variance component estimation of additive and dominance effects based on the traditional quantitative genetics model that partitions a genotypic value into breeding value and dominance deviation. The methodology will have two complimentary computing strategies for large numbers of individuals and markers, and the genomic prediction methods for have GBLUP and associated reliability for both training and validation data sets. Accuracies of the new methods will be evaluated using simulation data based a true dairy cattle SNP structure.

## Methods

### Genetic Model of SNP Markers and Mixed Model of Phenotypic Observations

The genetic model of SNP markers is an expansion of the additive model used in genomic evaluations [2], [4], [5] by adding a dominance component to the additive model. Using the traditional quantitative genetics model that partitions a genetic value into breeding value and dominance deviation under the assumption of Hardy-Weinberg equilibrium [7], the genetic value of each SNP marker can be expressed as:
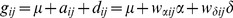
(1)where 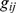 = genotypic value of SNP genotype 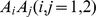, 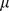 = common mean, 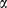 = average effect of gene substitution, 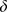 = dominance effect, *a_ij_* = 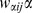 = breeding value, *d_ij_* = 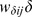 = dominance deviation, 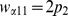, 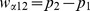, 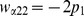, 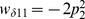, 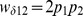, 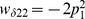, and where 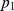 = frequency of 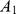 allele and 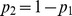 = frequency of 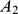. Note that gene substitution effect (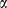) is a contrast of breeding values or a contrast of allelic effects, and dominance effect (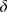) is a contrast of dominance deviations or a contrast of genotypic values (Text S1: Part A). In matrix notations, the genetic model of Equation 1 can be expressed as:



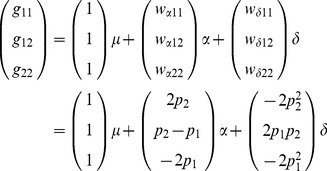
(2)The quantitative genetics model of Equation 2 has the interpretation of ‘breeding value’ for additive effects. Assuming equal allele frequency and using a reparameterized 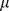, Equation 2 can achieve the (−1)-0-1 coding or 0-1-2 coding for additive effects and the 0-1-0 coding for dominance effects, but additive effects in those equal frequency models do not have the interpretation of ‘breeding value’ when the actual allele frequencies are unequal (Text S1: Part A). For each SNP marker, the variance of 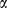 and the variance of 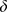 are assumed to be 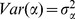 and 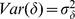, and the covariance between 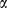 and 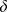 is assumed null. Let 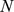 = the number of phenotypic observations, *q* = the number of individuals, *m* = the number of SNP markers, and *c* = the number of fixed effects. Based on Equation 2, the mixed model with SNP breeding values and dominance deviations can be expressed as:
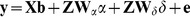
(3)where 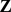 = 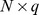 model matrix allocating phenotypic observations to SNP marker genotypes of individuals, 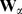 = 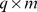 model matrix for gene substitution effects of SNP markers, 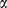 = column vector of gene substitution effects of SNP markers, 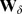 = 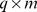 model matrix for dominance effects of SNP markers, 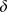 = column vector of dominance effects of SNP markers, 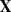 = 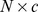 model matrix for fixed non-genetic effects such as herd-year-season in dairy cattle, and 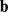 = vector of fixed effects. Assumptions for the first and second moments are: 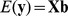, 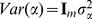, 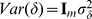, and 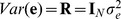, where 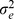 = residual variance, 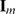 = 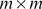 identity matrix, and 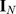 = 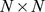 identity matrix. With the model and assumptions of Equations 1–3, methods for GBLUP and genomic variance component estimation using restricted maximum likelihood estimation (GREML) can be developed.

## Results and discussion

### Genomic Additive and Dominance Relationship Matrices

As the number of SNP markers increases, the values of the diagonal elements of 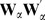 and 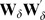 increase. Two methods to normalize the 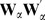 and 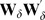 matrices can be used. The first method divides 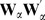 and 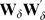 by the expected variance of the diagonal elements of each matrix (Definition I, [2]). The second method divides 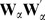 by the average of the diagonal elements of 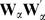 (Definition II, [4]), and we apply this method to 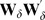 for defining dominance relationship matrix. In addition, we use a transformation to transform 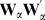 and 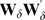 into correlation matrices so that off-diagonal elements are mathematically comparable, and we refer to this definition as Definition III and refer to the resulting correlation matrices as genomic additive and dominance correlation matrices. The additive correlations of Definition III are the genomic version of Wright’s coefficient of relationship [19]. Each of these three definitions of additive and dominance relationship or correlation matrices can be represented by two transformation matrices, 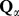 or 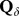. Let 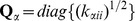 an 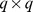 diagonal matrix, and 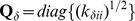 an 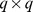 diagonal matrix, where 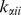 is the expected variance of the diagonal elements of 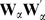 and 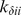 is the expected variance of the diagonal elements of 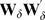 for Definition I (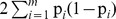 for additive relationships [2], and 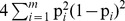 for dominance relationships, personal communication from P. VanRaden to Y. Da, March 3, 2013), 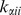 is the average of diagonal elements of 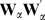 and 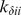 is the average diagonal elements of 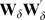 for Definition II, and 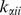 is the *i* ^th^ diagonal element of 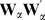 and 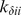 is the *i* ^th^ diagonal element of 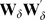 for Definition III. Then,
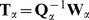
(4)

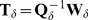
(5)


The additive relationship or correlation matrix (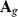) and dominance relationship or correlation matrix (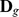) can be expressed as.
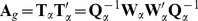
(6)

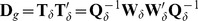
(7)


In Equations 6–7, subscript ‘*g*’ is used to distinguish 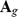 and 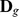 from the 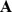 and 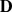 matrices calculated from pedigree data [20]. In addition to representing a number of definitions of genomic relationships, 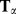 and 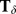 are used to define equivalent models to achieve computing efficiency.

### Two Equivalent Mixed Models, Two Sets of Complementary Formulations

With the 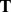 matrices of Equations 4–5, two equivalent mixed models with complementary computing advantages, Model 1 and Model 2, can be defined. Model 1 can be written as:
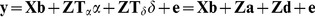
(8)

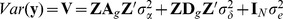
(9)where 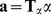 = genomic breeding values,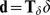 = genomic dominance deviations, 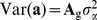, and 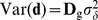. Model 2 can be rewritten as:



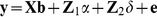
(10)

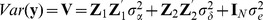
(11) (11)where 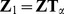 and 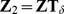. Model 1 of Equation 8 and Model 2 of Equation 10 have the same mathematical expectation, i.e., 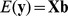. The two equivalent models of Equations 8–11 can generate four sets of formulations with identical results of GBLUP, reliability and GREML. Each model can use a conditional expectation (CE) or mixed model equations (MME) to calculate GBLUP. The CE set of Model 1 is the best for large number of markers (*m>q*) and the MME set of Model 2, to be referred as the QM set (QM meaning *q>m*), is the best for large number of individuals (*q>m*). The MME set of Model 1 (to be referred to as MQ, with MQ meaning *m>q*) has no computing advantage because the matrix size is twice as large as that of CE and requires the inverses of the relationship matrices. The CE set of Model 2 (CE2) also has no computational advantage because CE2 requires more memory than QM if *m>q*. These two sets (MQ and CE2) are not considered further. In the following, we focus on the CE and QM sets of solutions, where each set consists of GBLUP, reliability of GBLUP and GREML formulations. We first present these three types of formulations in each set, CE for *m>q* or QM for *q>m*, and then summarize the main features of the CE and QM sets.

### GBLUP-CE, Reliability and GREML-CE for *m>q*

The CE form of GBLUP from Model 1 can be calculated as:
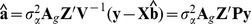
(12)

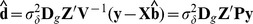
(13)where 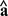 = GBLUP of breeding values, 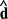 = GBLUP of dominance deviations, 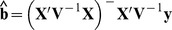 is best linear unbiased estimator (BLUE) of **b**, 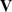 is defined by Equation 9, and



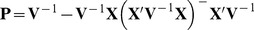
(14)We refer to the GBLUP of Equations 12–13 as GBLUP-CE. The GBLUP of genotypic values is calculated as 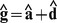. The reliability measures of 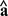, 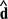 and 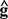 for individuals with phenotypic observations (individuals in training data set) are:
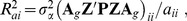


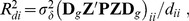


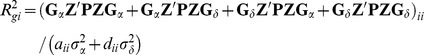
where 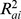 = the reliability of GBLUP of breeding values (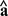) for individual *i*, 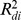 = the reliability of GBLUP for dominance deviations (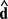) for individual *i*, 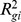 = the reliability of GBLUP for genotypic values (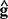), 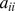 = diagonal element *i* of 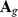, 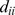 = diagonal element *i* of 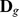, 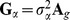, 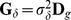, and 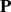 is given by Equation 14. Note that 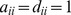 for Definition III but the 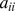 and 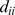 values generally are not ‘1′ for Definitions I and II. The average of 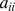 values and the average of 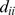
values are ‘1′ under Definitions II and III, and are expected to be ‘1′ under Definition I although the observed average 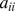 and 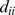
values under Definition I may deviate from ‘1′. For individuals without phenotypic observations (individuals in validation data set), formulations of GBLUP-CE and associated reliability measures are given in Text S1: Part B. GREML-CE via the EM type algorithm [20]–[22] are:



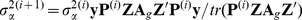
(15)

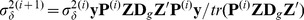
(16)

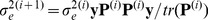
(17)


### GBLUP-QM, Reliability and GREML-QM for *q>m*

The mixed model equations for predicting SNP additive effects (**α**) and dominance effects (**δ**) based on Model 2 are:
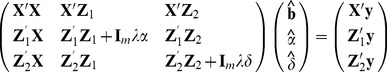
(18)where 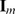 = 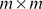 identity matrix, 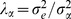 and 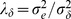. To reduce the size of Equation 18, equations for 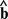 can be absorbed, and Equation 18 after the absorption reduces to:
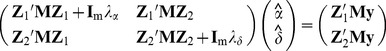
(19)where 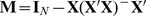. The GBLUP of breeding values and dominance deviations for all individuals with phenotypic observations can be calculated as:



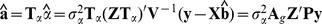
(20)

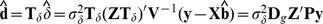
(21)where 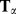 is defined by Equation 4, 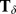 by Equation 5, and 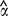 and 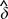 are solutions to Equation 16. We refer to the approach of Equations 19–21 as GBLUP-QM. The comparison between Equations 20–21 and Equations 12–13 shows that GBLUP-CE and GBLUP-QM are mathematically identical. Reliabilities of GBLUP-QM from Equations 19–21 are:
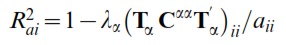


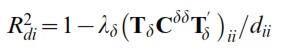


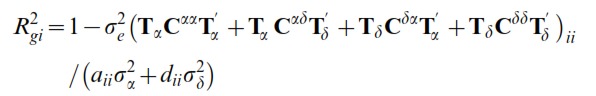
where **T**_α_ and **T**_δ_ are defined by Equations 4–5, and 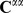, 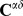, 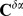
and 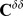 are submatrices that satisfy:



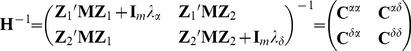
(22)For individuals without phenotypic observations (individuals in validation data set), formulations of GBLUP-QM and associated reliability measures are given in Text S1: Part B. GREML-QM formulations via EM type algorithm are:
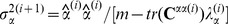
(23)

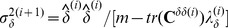
(24)

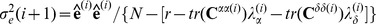
(25)where r is the rank of the coefficient matrix of Equation 18, 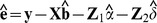 and 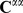 and 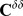 are defined by Equation 22.

### Heritability Estimates

Three heritability estimates can be obtained from estimates of variance components: additive heritability or heritability in the narrow sense (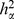), dominance heritability (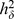), and the total heritability or heritability in the broad sense (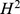). Let 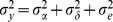 = phenotypic variance. Then, 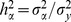, 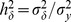, and 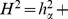 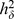. Note that the variances of additive and dominance effects (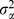 and 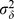) could be converted into the variances of breeding values and dominance deviations (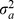 and 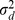) by 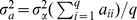 and 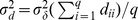 based on 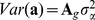 and 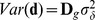 defined in Equation 9. However, this type of conversion practically is unnecessary because the average 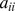 and 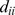
values are ‘1′ under Definitions II and III and are expected to be ‘1′ under Definition I of genomic additive and dominance relationships.

### Main Features of the CE and QM Formulations

The CE and QM sets of formulations for GBLUP, reliability and GREML are mathematically identical, offer identical results, and offer complimentary computing efficiency. The CE set is designed for *m>q* and is the best approach for using a large number of markers for GBLUP and GREML, while GBLUP-QM is designed for *q>m* and is the best approach for using a large number of individuals in GBLUP and GREML. A simple rule for choosing between CE and QM is: use CE if *q<2m* or vice versa. This is because the size of the **V** matrix to be inverted is *q* for CE (assuming one observation per individual) and the size of the MME coefficient matrix of Equation 19 is *2m* for QM so that **V** become easier to invert than the MME coefficient matrix of Equation 19 for *q<2m*. Both sets do not require the inversions of the additive and dominance relationship matrices. The CE set uses relationship matrices explicitly whereas the QM set does so implicitly. Both sets are invariant to the invertibility of 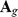 and 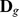, i.e., both sets are applicable to singular 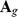 and 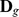, applicable to *m*>*q* where 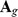 and 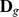 are generally invertible, and applicable to *q>m* where 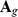 and 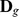 are non-invertible. The property of invariance to the invertibility of additive and dominance relationship matrices is a significant convenience because researchers do not have to require *m*>*q* and do not need to assess invertibility that is not guaranteed by *m>q*, e.g., the existence of identical twins results in non-invertible 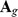 and 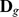.

### GBLUP for Validation Data Set, AI-REML, Computer Implementation

Formulations for GBLUP-CE and GBLUP-QM for individuals without phenotypic observations (individuals in validation data set) and reliability measures are given in Text S1: Part B. The EM type algorithm of Equations of 15–17 and 23–25 is known to be reliable but slow. The AI-REML algorithm [23]–[25] is fast but is not as reliable as EM type. The implementation of AI-REML for estimating additive, dominance and residual variance components is described in Text S1: Part C. All formulations for GBLUP, reliability, genomic relationships and GREML including AI-REML are implemented by the GVCBLUP package [26], which is freely available at http://animalgene.umn.edu.

### Accuracy of GREML and GBLUP for Additive and Dominance Heritabilities

Simulation study with known true values of genetic effects and parameters is an effective approach to evaluate the accuracy of a new methodology because the observed GBLUP and GREML estimates can be compared with the true values. We generated a large number of simulated data sets based on a true dairy cattle SNP structure of 1654 Holstein cows assuming true additive and dominance heritability levels of 0, 0.05, 0.15 and 0.30, and we applied seven SNP sets to the simulated data, 1K causal variants, 1K SNP, 2K SNP and causal variants, 3K, 7K 40K SNP markers, and 41K SNP markers and causal variants. Detailed information about these marker sets and the procedure to generate the simulation data are described in Text S1: Part D. For the sample size of 1654 individuals in the simulation study with seven causal and SNP marker sets, GREML were able to capture small effects that each accounted for only 0.00005–0.0003 of the phenotypic variance with high accuracy and were able to distinguish between high and low heritability levels. However, dominance GREML was less accurate and required higher density of SNP markers than additive GREML (Table S1). These results were encouraging given the rapid data growth in genomic selection [27]–[29] that could substantially increase the GREML accuracy for both additive and dominance effects over the accuracies observed with our sample size.

#### GREML accuracy of causal variants

**Figure 1:**
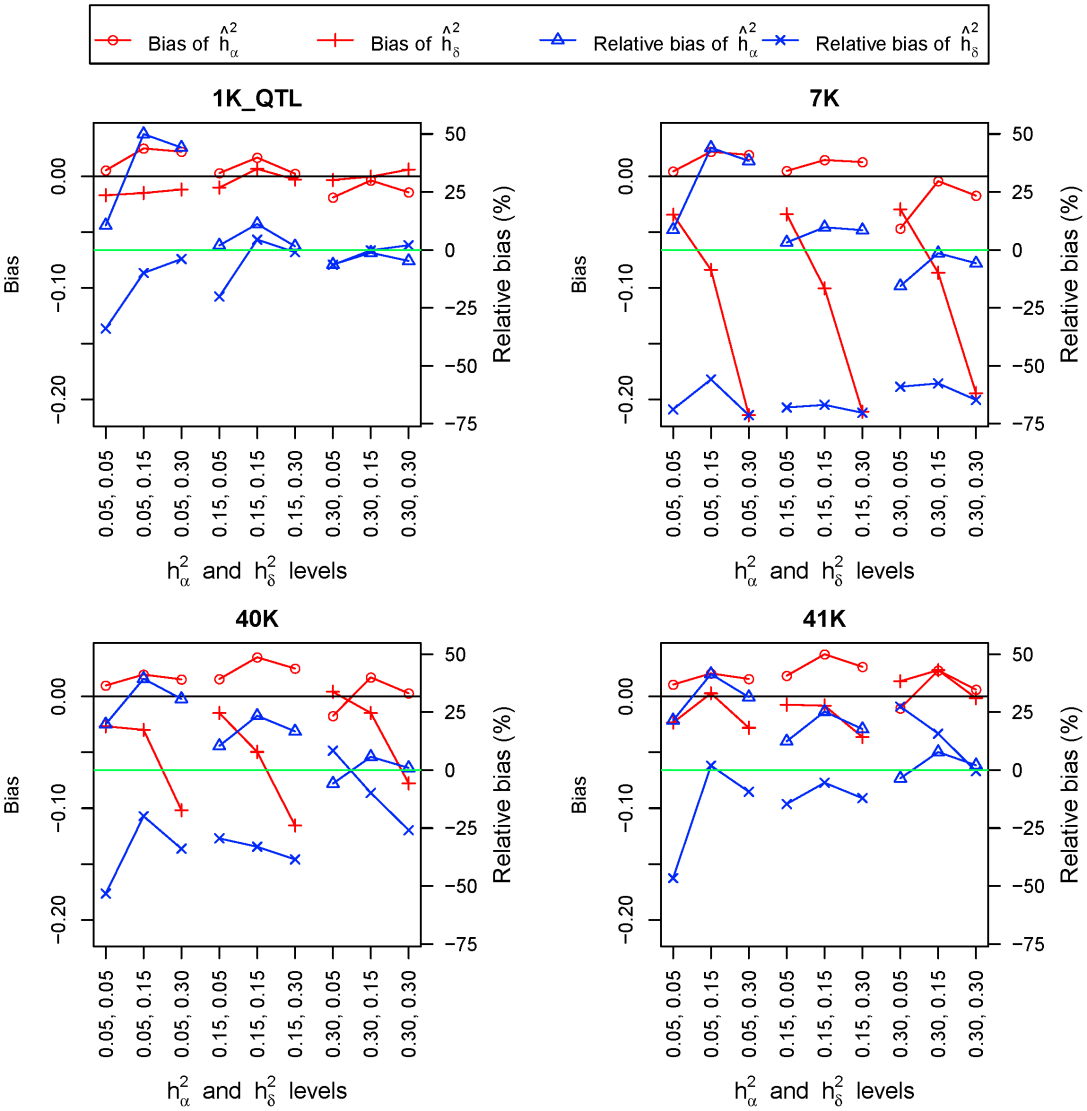
Bias and relative bias of GREML estimates of additive and dominance heritabilities. On the X-axis, heritiabilities of the top row are dominance heritabilities and those of the bottom row are additive heritabilities. (n = 10 repeats).

**Figure 2:**
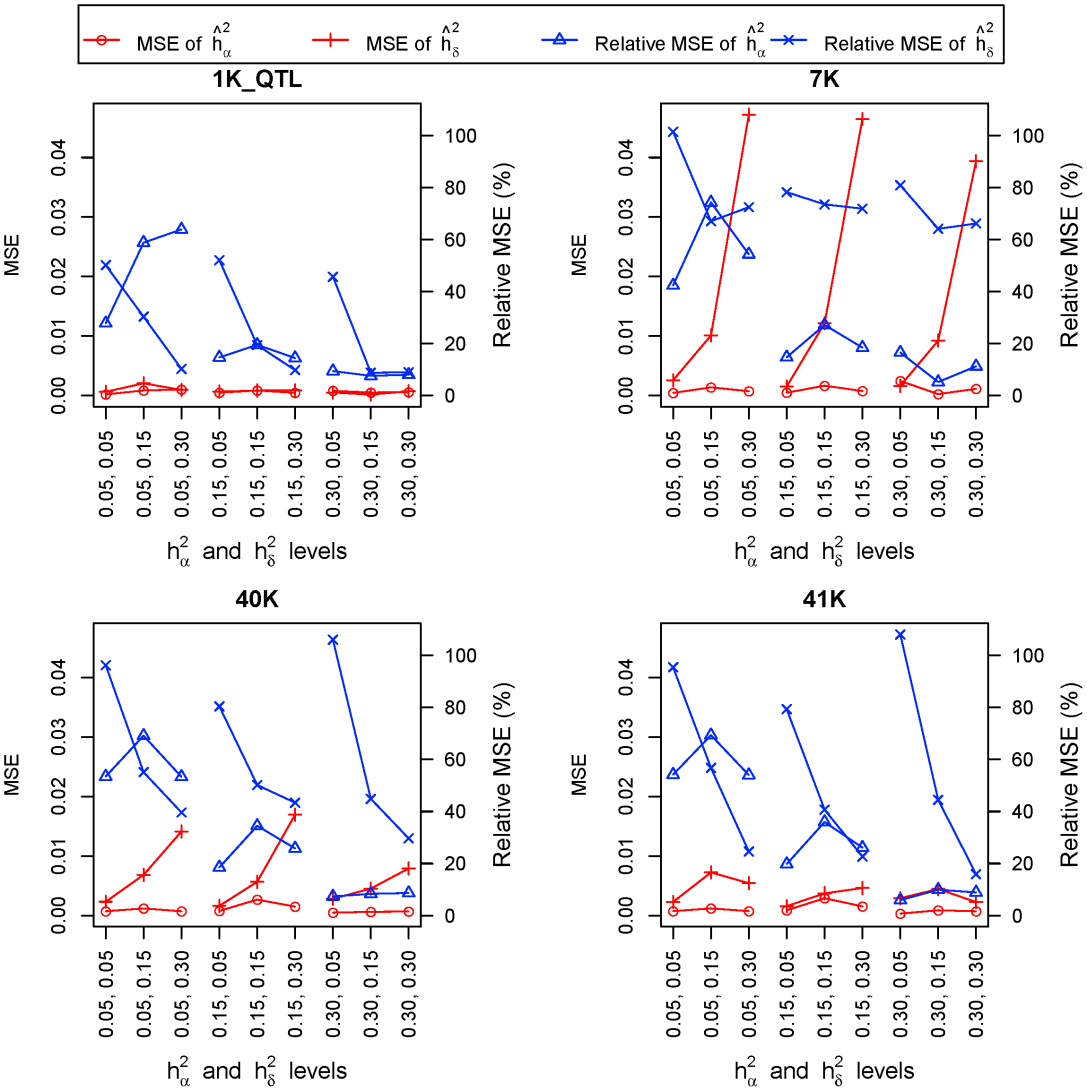
Mean square error (MSE) and relative MSE of GREML estimates of additive and dominance heritabilities. On the X-axis, heritiabilities of the top row are dominance heritabilities and those of the bottom row are additive heritabilities. (n = 10 repeats).

Causal SNP markers (1K_QTL, Table S1) had the best accuracy in almost all cases and had similar accuracies for both additive and dominance heritabilities except the case with 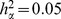 and 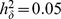, where the estimate of dominance heritability was 
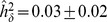
 and the estimate of additive heritability was 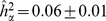. Adding linked SNP makers to the causal SNP (2K and 41K in Table S1) decreased GREML accuracy in most cases. Causal SNP markers had nearly unbiased estimates of heritabilities (Figure 1) and had the smallest MSE of heritability estimates (Figure 2). The bias and MSE of variance components had similar patterns as those for heritabilities (data not shown).

#### GREML accuracy of linked SNP markers

Linked SNP markers were less accurate than causal SNP markers in nearly all cases but were still highly accurate for estimating additive variance. For additive effects, GREML using the 40K and 41K SNP sets had a tendency of slightly overestimating additive heritabilities and variance components. For dominance effects, the marker densities in this simulation study, 1K_SNP, 3K, 7K and 40K, were all insufficient to achieve accurate estimates of dominance heritabilities and variance components, although the 40K set was able to distinguish between high and low dominance heritabilites. Accuracy of dominance GREML increased as the density of linked SNP marker increased from 1K_SNP to 40K, indicating that further increase in marker density over 40K could improve the accuracy of dominance GREML (Table S1).

#### GREML estimates for ‘0′ heritability

Estimating ‘0′ heritability generally is considerably more difficulty than estimating non-null heritability. Therefore, the accuracy in estimating ‘0′ heritability is a strong test for the accuracy of the GREML formulations. From the same simulation data set we generated above, we generated another set of simulation data requiring additive or dominance effects to be the only genetic effects such that 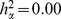 and/or 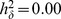 to test the performance of GREML when the true heritability and variance component for one or both effects were null. The causal variants (503_A and 503_D) again had the highest accuracy in estimating ‘0′ heritabilities and variance components, with average heritability estimates in the range 0–0.01 for additive heritability and 0–0.02 for dominance heritability (Table S2). The 1K SNP set with half causal variants and half inter-QTL SNP (503_A +503_D) was virtually as accurate as the causal variants of 503_A or 503_D. The 41K set also included the causal variants but were not as accurate as the 1K set and overestimated dominance heritability by 0.05 when the true dominance heritability was ‘0′. The 40K inter-QTL SNP markers had the same overestimates as the 41K. The results of the 1K, 40K and 41K SNP sets showed that a large number of linked SNP markers decreased the GREML accuracy when the true dominance heritability was null. Overall, the GREML formulations were surprisingly accurate in estimating null additive and dominance heritabilities except the 40K and 41K marker sets for null dominance heritability.

#### Accuracy of GBLUP for breeding values, dominance deviations and genetic values

**Figure 3:**
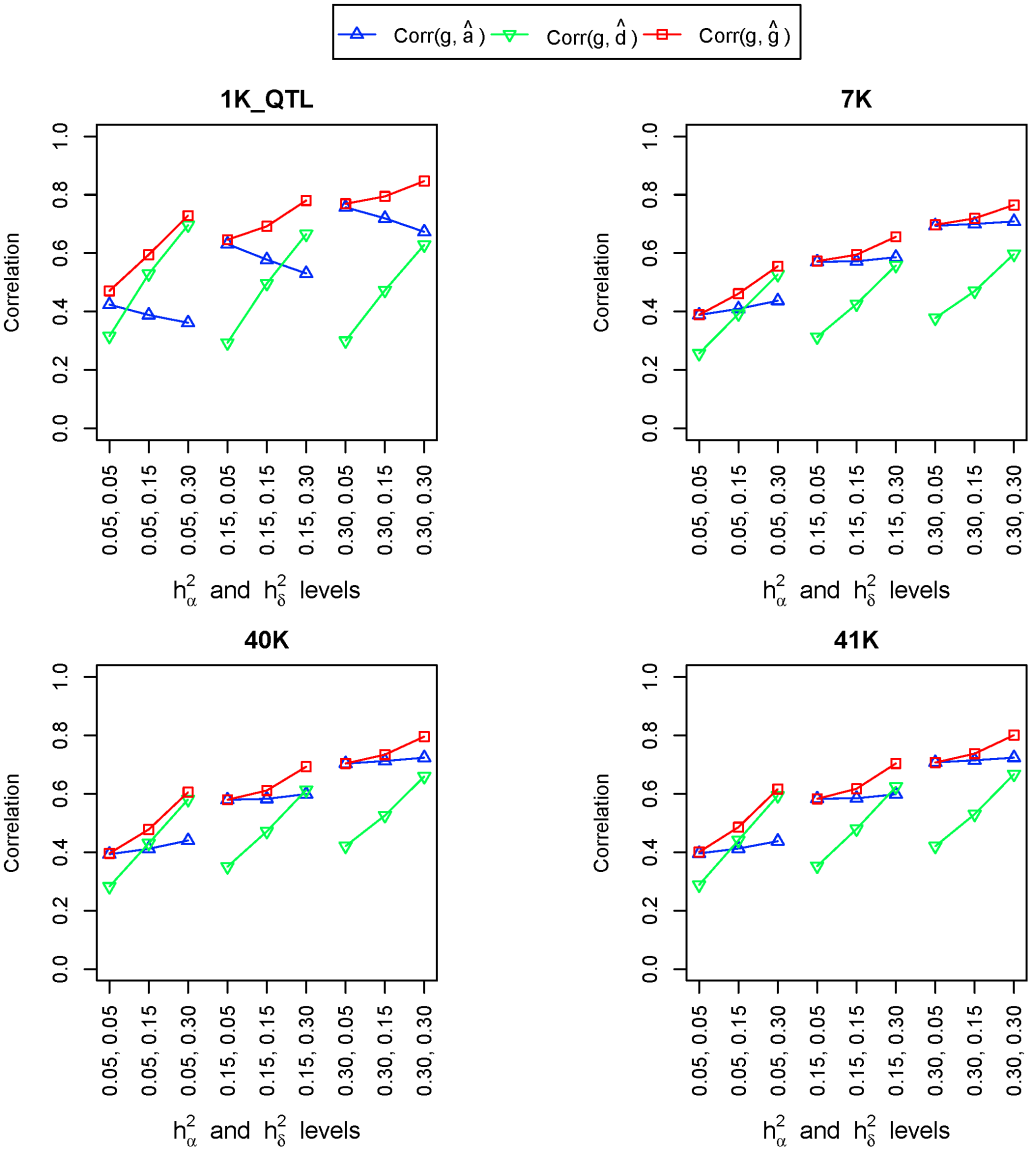
Correlation between the true genotypic values and GBLUP of breeding values, dominance deviations and genetic values. 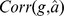
 is the correlation between true genotypic values and GBLUP of breeding values, 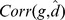 is the correlation between true genotypic values and GBLUP of dominance deviations, and 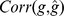 is the correlation between true genotypic values and GBLUP of genotypic values. On the X-axis, heritiabilities of the top row are dominance heritabilities and those of the bottom row are additive heritabilities. (n = 10 repeats).

GBLUP of genotypic values (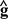) and GBLUP of breeding values (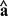) were less sensitive to marker density than GREML. GBLUP of dominance deviations (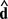) was sensitive to marker density as was dominance GREML. Observed and expected accuracies all increased as heritability levels increased. The benefit of using 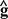 over 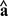 or 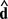 for predicting the total genotypic values increased as dominance heritability increased for a given additive heritability except the case 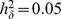 (Figure 3).

#### GBLUP accuracy of causal variants and linked SNP markers

Causal variants had the best GBLUP accuracy for 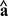, 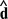 and 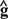, but the accuracy for 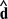 was lower than that for 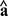 and 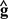, unlike additive and dominance GREML that had similar accuracies using causal variants. The difference in observed GBLUP accuracy between 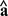 and 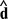 was the largest for low additive and dominance heritabilities at 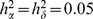 with 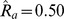 and 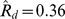, and was the smallest for high heritabilities at 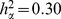 and 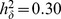 with 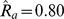 and 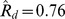. These results indicated that dominance GBLUP could be considerably more difficult than additive GBLUP for low dominance heritabilities. Observed accuracy of 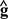 (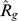) was higher than that of for 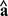 (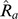) i.e., 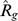
>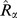 for all heritability levels in this simulation study except the case 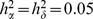 (Table S3).

For various densities of inter-QTL SNP markers ranging from 3K, 7K to 40K, 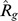 and 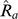 were relatively unchanged within each combination of additive and dominance heritability levels, indicating that increasing SNP density over 3K would achieve little improvement in 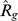
and 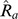. For the 1K_SNP, 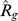
and 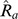 were lower than the 3K, 7K and 40K by about 0.05. In contrast, 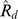 was similar for the 7K and 40K, had substantial decrease for the 1K_SNP and 3K, and was considerably lower than 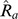 across all heritability combinations. These results indicated that dominance GBLUP required higher density of SNP markers and was more difficult than additive GBLUP (Table S3).

Adding linked SNP makers to causal variants (1K_QTL +1K_SNP, 1K_QTL +40K) had lower observed accuracies than causal variants alone. The decrease in 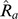 was 0.03 for adding the 1K_SNP to the 1K_QTL and was 0.06 for adding the 40K to the 1K_QTL. The decreases in 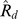
were even larger, 0.06 and 0.13, respectively. These decreases were relatively constant across heritability levels (Table S3). However, any marker set with causal variants, the 2K or 41K, was more accurate than linked SNP only, the 1K_SNP, 3K, 7K or 40K.

#### Predicted and observed GBLUP accuracies

Predicted accuracy for breeding values (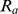) and for genotypic values (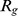) agreed well with the observed accuracies (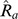
and 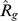) across all heritability levels used in this study. For dominance deviations, predicted accuracy (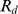) and observed accuracy (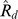) agreed well except 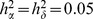, where 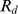 was substantially lower than observed accuracies. In real data sets, observed accuracies measured by 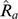, 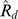
and 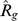 are unavailable. The good agreements between predicted and observed accuracies indicated that predicted accuracy could reliably represent the observed accuracy in real data.

### Comparison of Genomic Additive and Dominance Relationships with Expected Relationships

**Figure 4:**
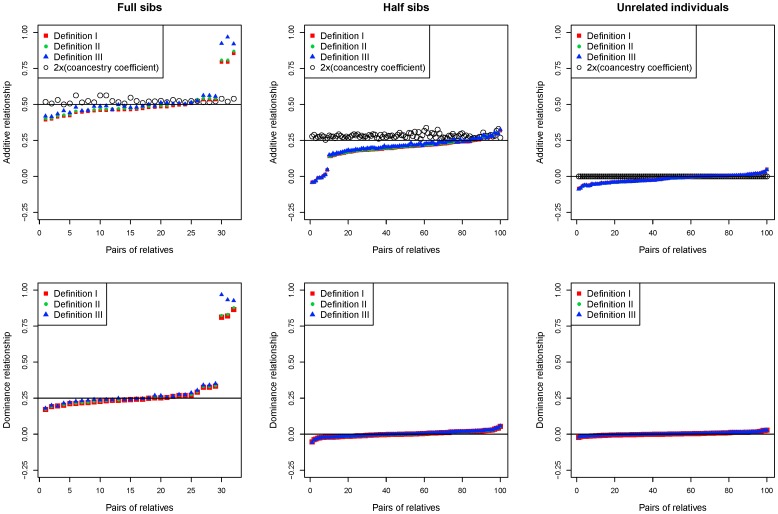
Genomic additive and dominance relationships among full-sibs, half-sibs and unrelated individuals.

For genomic additive and dominance relationships, Definitions I-III had nearly identical results. The 1K, 3K, 7K and 41K marker sets had similar results of relationships (data not shown). For the 41K results with the removal of three full-sib outliers and nine half-sib outliers, additive and dominance relationships agreed well with theoretical expectations (Figure 4). For full-sibs, genomic additive and dominance relationships were nearly identical to theoretical expectations. Average genomic additive relationships was 0.471 for Definition I, 0.478 for Definition II, and 0.488 for Definition III, while the mean value of pedigree coancestry coefficients for full-sibs was 0.262, i.e., genomic additive relationships were about twice as large as pedigree coancestry coefficients. The mean dominance correlation for full-sibs was 0.245 for Definition I, 0.248 for Definition II and 0.254 for Definition III, compared to the expected full-sib dominance correlation of 0.25 assuming no inbreeding. The 1654 cows used in this comparison of genomic and pedigree relationships in fact were all related [30]. Therefore, the true full-sib dominance relationships should have been above 0.25. For half-sibs, Definitions I-III had mean additive relationship of 0.213–0.221 and the average of ‘2**×**(pedigree coancestry coefficient)’ was 0.282. Genomic dominance relationships were null for half-sibs and unrelated individuals, and genomic additive relationships for unrelated individuals were also null, as expected (Figure 4). The observed similarity between Definitions I-III likely was due to the underlying similarity of the three definitions: Definition I and II are the same if the expected and observed SNP variances are the same, and Definitions II and III are the same if all diagonal elements are the same. Definitions I and II make slightly less modification to the original mixed model of Equation 3, whereas off-diagonal elements of Definition III as measures of genomic relatedness among individuals are mathematically comparable.

Genomic relationships have a distinct advantage over pedigree relationships: the calculation of genomic relationships does not need to know the pedigree. This advantage is important for assessing relatedness among individuals in species where pedigree information is unavailable or difficult to collect such as in wildlife species. Two important differences exist between relationships based on markers and relationships based on pedigree information. The first difference is that marker density affects the invertibility of genomic relationship matrices, which are non-invertible when *q>m*. In contrast, pedigree relationship matrices are positive definite in the absence of identical twins. Our cattle data showed that the invertibility of a genomic relationship matrix should not affect the use of genomic relationships as measures of genomic relatedness among individuals, because the genomic relationships calculated from genomic relationship matrices that were invertible or non-invertible had nearly identical values that were consistent with theoretical expectations (data not shown). The additive and dominance relationship matrices were non-invertible for the 1K SNP set, and were invertible for the 3K, 7K and 41K sets after removing a potentially identical twin or duplicated individual. The second difference is the range of relationship values. Genomic relationships by Definitions I-III could take negative values whereas pedigree relationships are non-negative. However, no negative values were observed for full-sib genomic relationships. Negative genomic additive relationships with small absolute values near ‘0′ were observed for unrelated individuals and some half-sibs, and negative dominance relationships with small absolute values near ‘0′ were observed among half-sib (Figure 4). In all those situations, the expected relationships were ‘0′. Therefore, negative genomic relationships close to ‘0′ could be interpreted as no correlation. It remains to be seen whether genomic relationship measures could detect true ‘negative genomic correlations’ (if such correlations exist) that are impossible to detect using pedigree information.

### Effect of Genomic Relationship Definitions on GBLUP and GREML Accuracies

Simulation results showed that the methods to normalize 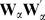 and 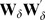 (Definitions I and II of genomic relationships) had no effect on GBLUP accuracy, i.e., the original mixed model was just as accurate, as shown by the 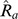 and 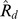
values (Table S4). Definition III of genomic relationships had the same accuracy as Definitions I-II for breeding values and had slightly lower accuracy for dominance deviations for one case only at 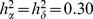, with 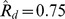 for Definition III and 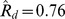 for Definitions I and II (Table S4). For GREML, normalization or transformation of the 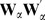 and 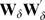 matrices was necessary. Without such normalization or transformation, diagonal values in 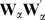 and 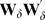
increased and estimates of variance components decreased as the number of SNP markers increased regardless of the true heritability level, so that hertitability estimates based on such variance component estimates became meaningless, as shown by the comparison of GREML estimates and the corresponding heritability estimates in Table S4. For GREML estimation of additive and dominance variances, Definitions I-III had similar estimates that were consistent with the true values.

### Random and Directional Dominance Effects

Random additive and dominance effects with zero means were assumed in the simulation study reported in the section of Results. Under these assumptions, dominance effects were more difficult to predict and estimate in two aspects: the current densities of inter-QTL SNP markers up to 40K were insufficient to achieve accuracies comparable to those for additive effects, and causal variants had lower accuracy of dominance GBLUP than the accuracy of additive GBLUP, although causal variants had similar accuracy for estimating additive and dominance variance components. The simulation results indicated that the number of SNP markers needed in the absence of causal variants would be considerably greater than 40K to achieve accuracies of dominance GBLUP and GREML comparable to the accuracies of additive GBLUP and GREML. High density of SNP markers could also compensate the lower accuracies of causal variants, whether or not causal variants were among the SNP markers. The simulation data set assuming positive dominance deviation for each heterozygous genotype (Text S1: Part D) showed that dominance GBLUP had similar accuracies to additive GBLUP (Table S5).

Taken all evidence together, genomic prediction and variance component estimation of dominance effects was more difficult than those of additive effects in populations where additive and dominance effects had similar distributions and heritabilities but could achieve similar accuracies as those for additive effects if heterosis exists.

### An Application to Estimate Genomic Additive and Dominance Heritabilities in a Swine Population

We applied our methodology to a publically available swine genomics data set with anonymous genome-wide SNP markers and phenotypes with the SNP locations and true trait names masked [31] to compare genomic additive heritability with the reported heritability estimated using pedigree information and to explore whether the swine phenotypes had dominance effects. The data set included 3534 animals from a single PIC nucleus pig line with genotypes from the Illumina PorcineSNP60 chip [32]. Genotyped animals had phenotypes for five purebred traits (phenotypes in a single nucleus line), with additive heritability estimated from pedigree data ranging from 0.07 to 0.62 (Table 1). Genotypes were filtered by requiring minor allele frequency (MAF) >0.001 and proportion of missing SNP genotypes <0.100. Markers on the X or Y chromosome were excluded. The total number of available autosome markers used in our analysis was 52,842, with missing genotypes imputed using software AlphaImpute [33]. The results showed that estimates of genomic additive heritability of 0.22–0.38 were substantially lower than the pedigree estimates of 0.38–0.62 for traits 3–4, the genomic additive heritability (0.27) was higher than the pedigree estimate (0.16) for trait 2, and was in agreement with the pedigree estimate for trait 1, 0.03 versus 0.07. Only traits 3 and 5 had small dominance heritabiities, 0.07 for trait 3 and 0.05 for trait 5. The genomic estimates reported here provide useful information to breeders about the underlying true genetic factors and about the potential true heritability levels of the five traits.

**Table 1:** Estimated genomic additive and dominance heritabilities from a swine nucleus line.

	Trait 1	Trait 2	Trait 3	Trait 4	Trait 5
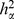	0.03 (0.07*)	0.27 (0.16)	0.22 (0.38)	0.35 (0.58)	0.38 (0.62)
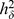	7.22×10^−7^	0.02	0.07	0.01	0.05
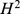	0.03	0.29	0.29	0.36	0.44

* Value in each () is the pedigree-based heritability estimate [31].

## Conclusions

The genomic model based on the partition of a genotypic value into breeding value and dominance deviation with additive and dominance relationship matrices calculated using SNP markers parallels the traditional quantitative genetics model that calculates additive and dominance relationships using pedigree information. The GREML and GBLUP methods based on equivalent models with complementary computing advantages and identical mathematical results provide an efficient approach for the genomic estimation of variance components and heritabilities and for the genomic prediction of additive and dominance effects using SNP markers. These methods were able to capture small additive and dominance effects and were able to differentiate different levels of additive and dominance heritabilities. GBLUP of total genetic value that includes additive and dominance effects can be an effective tool to predict an individual’s total genetic potential for a phenotype.

## Supporting Information

Table S1 GREML estimates of variance components and heritabilities of additive and dominance effects (mean ± standard deviation, n = 10 repeats).
(PDF)

Table S2 GREML estimates and GBLUP accuracy for simulation data with additive or dominance effects only (mean ± standard deviation, n = 10 repeats).
(PDF)

Table S3 GBLUP Accuracies for breeding values, dominance deviations and genotypic values (mean ± standard deviation, n = 10 repeats).
(PDF)

Table S4 GREML estimates of variance components and GBLUP accuracies with and without genomic relationships for phenotypes with additive and dominance effects of 1006 QTL (mean ± standard deviation, n = 10 repeats).
(PDF)

Table S5 GBLUP accuracies in simulation data assuming random additive effects and directional dominance effects values (mean ± standard deviation, n = 10 repeats).
(PDF)

Text S1 **Proofs, formulations and simulation study.** Part A: Derivations for the traditional quantitative genetics model of SNP markers with unequal and equal allele frequencies. Part B: Genomic prediction of additive and dominance effects for individuals without phenotypic observations. Part C: AI-REML implementation. Part D: Simulation study to evaluate GREML and GBLUP accuracies.
(PDF)
