# Supplementary material for: Mixed Model Methods for Genomic Prediction and Variance Component Estimation of Additive and Dominance Effects Using SNP Markers
Source: PLoS One. 2014 Jan 30;9(1):e87666. doi: 10.1371/journal.pone.0087666 (PMC3907568; doi:10.1371/journal.pone.0087666)
Supplement: Table S1 — GREML estimates of variance components and heritabilities of additive and dominance effects (mean ± standard deviation, n = 10 repeats). (PDF) [file pone.0087666.s001.pdf]

Table S1 GREML estimates of variance components and heritabilities of additive and dominance effects (mean ± standard deviation, *n*=10 repeats)

h^2 and σ^2	SNP type and density	$\hat{h}_\alpha^2$	$\hat{h}_\delta^2$	$\hat{\sigma}_\alpha^2$	$\hat{\sigma}_\delta^2$
h_α^2=0.05	1K_QTL	0.06±0.01	0.03±0.02	0.06±0.02	0.04±0.02
h_δ^2=0.05	1K_SNP	0.04±0.01	0.01±0.02	0.04±0.02	0.01±0.02
σ_α^2=0.06	2K (1K_QTL+1K_SNP)	0.06±0.02	0.03±0.02	0.07±0.02	0.03±0.02
σ_δ^2=0.06	3K	0.05±0.02	0.01±0.03	0.05±0.02	0.01±0.04
	7K	0.05±0.02	0.02±0.04	0.06±0.02	0.02±0.04
	40K	0.06±0.02	0.02±0.04	0.07±0.03	0.03±0.04
	41K (40K+1K_QTL)	0.06±0.02	0.03±0.04	0.07±0.03	0.03±0.05
h_α^2=0.05	1K_QTL	0.07±0.02	0.14±0.04	0.09±0.02	0.17±0.06
h_δ^2=0.15	1K_SNP	0.05±0.02	0.01±0.01	0.06±0.03	0.01±0.02
σ_α^2=0.06	2K (1K_QTL+1K_SNP)	0.08±0.02	0.15±0.05	0.10±0.02	0.18±0.07
σ_δ^2=0.19	3K	0.07±0.03	0.03±0.03	0.08±0.04	0.04±0.04
	7K	0.07±0.03	0.07±0.06	0.09±0.04	0.08±0.07
	40K	0.07±0.03	0.12±0.08	0.09±0.04	0.15±0.10
	41K (40K+1K_QTL)	0.07±0.03	0.15±0.09	0.09±0.04	0.19±0.11
h_α^2=0.05	1K_QTL	0.07±0.02	0.29±0.03	0.11±0.04	0.45±0.05
h_δ^2=0.30	1K_SNP	0.05±0.02	0.02±0.02	0.07±0.03	0.03±0.02
σ_α^2=0.08	2K (1K_QTL+1K_SNP)	0.07±0.02	0.29±0.03	0.11±0.03	0.45±0.05
σ_δ^2=0.46	3K	0.06±0.02	0.05±0.03	0.10±0.03	0.07±0.04
	7K	0.07±0.02	0.09±0.04	0.11±0.03	0.13±0.06
	40K	0.07±0.02	0.20±0.06	0.10±0.03	0.31±0.10
	41K (40K+1K_QTL)	0.07±0.02	0.27±0.07	0.10±0.03	0.42±0.11
h_α^2=0.15	1K_QTL	0.15±0.02	0.04±0.02	0.19±0.03	0.05±0.03
h_δ^2=0.05	1K_SNP	0.09±0.02	0.01±0.02	0.11±0.02	0.02±0.02
σ_α^2=0.19	2K (1K_QTL+1K_SNP)	0.15±0.02	0.05±0.02	0.19±0.03	0.06±0.02
σ_δ^2=0.06	3K	0.13±0.02	0.02±0.03	0.16±0.03	0.03±0.03
	7K	0.15±0.02	0.02±0.02	0.19±0.03	0.02±0.02
	40K	0.17±0.02	0.04±0.04	0.21±0.03	0.04±0.05
	41K (40K+1K_QTL)	0.17±0.02	0.04±0.04	0.21±0.03	0.05±0.05
h_α^2=0.15	1K_QTL	0.17±0.02	0.16±0.03	0.24±0.04	0.22±0.04
h_δ^2=0.15	1K_SNP	0.10±0.03	0.01±0.01	0.15±0.04	0.01±0.02
σ_α^2=0.21	2K (1K_QTL+1K_SNP)	0.17±0.03	0.15±0.04	0.24±0.03	0.22±0.05
σ_δ^2=0.21	3K	0.14±0.04	0.01±0.02	0.21±0.05	0.02±0.02
	7K	0.16±0.04	0.05±0.05	0.24±0.06	0.07±0.07
	40K	0.19±0.04	0.10±0.06	0.27±0.06	0.14±0.08
	41K (40K+1K_QTL)	0.19±0.04	0.14±0.06	0.27±0.06	0.20±0.09
h_α^2=0.15	1K_QTL	0.15±0.02	0.30±0.03	0.28±0.04	0.54±0.06
h_δ^2=0.30	1K_SNP	0.11±0.02	0.01±0.01	0.19±0.04	0.02±0.02
σ_α^2=0.27	2K (1K_QTL+1K_SNP)	0.16±0.03	0.30±0.04	0.29±0.05	0.54±0.07
σ_δ^2=0.55	3K	0.14±0.02	0.04±0.03	0.25±0.04	0.07±0.06
	7K	0.16±0.02	0.09±0.04	0.30±0.04	0.16±0.08
	40K	0.18±0.03	0.18±0.06	0.32±0.05	0.34±0.11
	41K (40K+1K_QTL)	0.18±0.03	0.26±0.06	0.32±0.05	0.48±0.10

h_α^2=0.30	1K_QTL	0.28±0.02	0.05±0.02	0.42±0.04	0.07±0.03
h_δ^2=0.05	1K_SNP	0.17±0.02	0.01±0.02	0.26±0.03	0.02±0.03
σ_α^2=0.46	2K (1K_QTL+1K_SNP)	0.29±0.02	0.05±0.04	0.44±0.04	0.08±0.06
σ_δ^2=0.08	3K	0.22±0.02	0.01±0.02	0.34±0.03	0.02±0.03
	7K	0.25±0.02	0.02±0.03	0.38±0.03	0.03±0.04
	40K	0.28±0.01	0.05±0.05	0.43±0.03	0.08±0.08
	41K (40K+1K_QTL)	0.29±0.01	0.06±0.05	0.44±0.03	0.10±0.08
h_α^2=0.30	1K_QTL	0.30±0.02	0.15±0.01	0.53±0.05	0.27±0.03
h_δ^2=0.15	1K_SNP	0.19±0.01	0.01±0.02	0.34±0.02	0.02±0.03
σ_α^2=0.55	2K (1K_QTL+1K_SNP)	0.31±0.02	0.15±0.03	0.56±0.05	0.27±0.05
σ_δ^2=0.27	3K	0.25±0.02	0.02±0.02	0.46±0.04	0.04±0.04
	7K	0.30±0.02	0.06±0.04	0.54±0.03	0.12±0.08
	40K	0.32±0.02	0.14±0.07	0.58±0.04	0.25±0.12
	41K (40K+1K_QTL)	0.32±0.02	0.17±0.06	0.59±0.04	0.32±0.11
h_α^2=0.30	1K_QTL	0.29±0.02	0.31±0.03	0.69±0.06	0.74±0.07
h_δ^2=0.30	1K_SNP	0.18±0.02	0.02±0.01	0.45±0.05	0.05±0.03
σ_α^2=0.75	2K (1K_QTL+1K_SNP)	0.30±0.02	0.31±0.03	0.72±0.06	0.75±0.08
σ_δ^2=0.75	3K	0.24±0.03	0.04±0.02	0.59±0.07	0.11±0.04
	7K	0.28±0.03	0.11±0.04	0.69±0.08	0.26±0.10
	40K	0.30±0.03	0.22±0.04	0.74±0.08	0.55±0.11
	41K (40K+1K_QTL)	0.31±0.03	0.30±0.05	0.75±0.08	0.73±0.12

h_α^2 = true additive heritability, h_δ^2 = true dominance heritability, σ_α^2 = true additive variance, σ_δ^2 = true dominance variance, $\hat{h}_\alpha^2$ = estimated additive heritability, $\hat{h}_\delta^2$ = estimated dominance heritability, $\hat{\sigma}_\alpha^2$ = estimated additive variance, σ_δ^2 = estimated dominance variance.
