# Supplementary material for: Mixed Model Methods for Genomic Prediction and Variance Component Estimation of Additive and Dominance Effects Using SNP Markers
Source: PLoS One. 2014 Jan 30;9(1):e87666. doi: 10.1371/journal.pone.0087666 (PMC3907568; doi:10.1371/journal.pone.0087666)
Supplement: Table S2 — GREML estimates and GBLUP accuracy for simulation data with additive or dominance effects only (mean ± standard deviation, n = 10 repeats). (PDF) [file pone.0087666.s002.pdf]

Table S2 GREML estimates and GBLUP accuracy for simulation data with additive or dominance effects only (mean ± standard deviation, *n*=10 repeats)

h^2 and σ^2	SNP Density	$\hat{\sigma}_\alpha^2$	$\hat{h}_\alpha^2$	R_a	$\hat{R}_a$	$\hat{\sigma}_\delta^2$	$\hat{h}_\delta^2$	R_d	$\hat{R}_d$
h_α^2=0.00	503_A	0.00±0.01	0.00±0.01	0.08±0.13	--	0.01±0.01	0.01±0.01	0.18±0.17	--
h_δ^2=0.00	503_D	0.00±0.01	0.00±0.01	0.06±0.14	--	0.00±0.01	0.00±0.01	0.09±0.12	--
σ_α^2=0.00	1K (503_A+503_D)	0.00±0.01	0.00±0.01	0.07±0.13	--	0.01±0.01	0.01±0.01	0.14±0.13	--
σ_δ^2=0.00	3K	0.01±0.01	0.01±0.01	0.08±0.14	--	0.01±0.01	0.01±0.01	0.06±0.09	--
	7K	0.01±0.02	0.01±0.02	0.08±0.15	--	0.01±0.02	0.01±0.02	0.09±0.10	--
	40K	0.01±0.02	0.01±0.02	0.06±0.14	--	0.05±0.05	0.05±0.05	0.19±0.15	--
	41K (40K + 1K)	0.01±0.02	0.01±0.02	0.06±0.14	--	0.05±0.04	0.05±0.04	0.19±0.14	--
h_α^2=0.05	503_A	0.06±0.01	0.05±0.01	0.51±0.03	0.56±0.04	0.01±0.01	0.01±0.01	0.18±0.17	--
h_δ^2=0.00	503_D	0.03±0.01	0.03±0.01	0.40±0.08	0.37±0.04	0.01±0.01	0.01±0.01	0.17±0.15	--
σ_α^2=0.05	1K (503_A+503_D)	0.06±0.01	0.06±0.01	0.47±0.03	0.52±0.05	0.02±0.02	0.02±0.02	0.18±0.16	--
σ_δ^2=0.00	3K	0.06±0.02	0.06±0.02	0.44±0.06	0.43±0.04	0.01±0.02	0.02±0.02	0.11±0.11	--
	7K	0.07±0.02	0.06±0.02	0.43±0.05	0.44±0.04	0.03±0.02	0.02±0.02	0.15±0.11	--
	40K	0.07±0.03	0.07±0.03	0.42±0.06	0.45±0.04	0.05±0.04	0.05±0.03	0.21±0.12	--
	41K (40K + 1K)	0.07±0.03	0.07±0.03	0.43±0.06	0.45±0.04	0.05±0.04	0.05±0.03	0.21±0.12	--
h_α^2=0.15	503_A	0.17±0.03	0.14±0.02	0.69±0.03	0.72±0.02	0.01±0.01	0.01±0.01	0.15±0.15	--
h_δ^2=0.00	503_D	0.08±0.02	0.07±0.02	0.56±0.06	0.49±0.02	0.01±0.01	0.01±0.01	0.15±0.14	--
σ_α^2=0.18	1K (503_A+503_D)	0.17±0.03	0.15±0.02	0.63±0.03	0.67±0.02	0.01±0.02	0.01±0.02	0.11±0.16	--
σ_δ^2=0.00	3K	0.14±0.04	0.12±0.03	0.55±0.04	0.58±0.02	0.02±0.02	0.02±0.02	0.13±0.12	--
	7K	0.17±0.04	0.14±0.03	0.56±0.04	0.58±0.02	0.03±0.05	0.03±0.04	0.12±0.16	--
	40K	0.18±0.04	0.16±0.03	0.56±0.04	0.59±0.02	0.05±0.07	0.04±0.06	0.16±0.16	--
	41K (40K + 1K)	0.19±0.04	0.16±0.03	0.57±0.04	0.60±0.02	0.05±0.07	0.04±0.06	0.16±0.17	--
h_α^2=0.30	503_A	0.40±0.05	0.29±0.02	0.81±0.01	0.82±0.01	0.00±0.01	0.00±0.01	0.09±0.12	--
h_δ^2=0.00	503_D	0.18±0.03	0.13±0.02	0.66±0.03	0.58±0.01	0.00±0.01	0.00±0.01	0.04±0.09	--
σ_α^2=0.43	1K (503_A+503_D)	0.41±0.06	0.30±0.03	0.76±0.02	0.78±0.01	0.00±0.00	0.00±0.00	0.00±0.00	--
σ_δ^2=0.00	3K	0.33±0.05	0.24±0.03	0.67±0.02	0.68±0.01	0.02±0.03	0.01±0.02	0.10±0.12	--
	7K	0.38±0.06	0.27±0.03	0.68±0.02	0.70±0.01	0.00±0.00	0.00±0.00	0.01±0.02	--
	40K	0.42±0.05	0.30±0.03	0.68±0.02	0.71±0.01	0.01±0.02	0.01±0.01	0.06±0.08	--
	41K (40K + 1K)	0.43±0.06	0.31±0.03	0.69±0.02	0.71±0.01	0.01±0.01	0.01±0.01	0.06±0.07	--
h_α^2=0.00	503_A	0.00±0.00	0.00±0.00	0.09±0.12	--	0.01±0.01	0.01±0.01	0.12±0.13	0.12±0.02
	503_D	0.00±0.00	0.00±0.00	0.06±0.11	--	0.04±0.02	0.04±0.01	0.39±0.14	0.44±0.03

h_δ^2=0.05	1K (503_A+503_D)	0.00±0.00	0.00±0.00	0.08±0.09	--	0.04±0.03	0.04±0.03	0.56±0.03	0.57±0.02
	3K	0.01±0.01	0.01±0.01	0.11±0.13	--	0.01±0.01	0.01±0.01	0.10±0.07	0.18±0.03
σ_α^2=0.00	7K	0.00±0.01	0.01±0.01	0.08±0.10	--	0.02±0.03	0.02±0.03	0.10±0.12	0.21±0.03
σ_δ^2=0.05	40K	0.00±0.01	0.00±0.00	0.08±0.09	--	0.04±0.04	0.04±0.04	0.17±0.14	0.23±0.02
	41K (40K + 1K)	0.00±0.01	0.00±0.00	0.08±0.09	--	0.05±0.05	0.04±0.05	0.18±0.14	0.24±0.02
h_α^2=0.00	503_A	0.01±0.01	0.00±0.01	0.09±0.15	--	0.01±0.01	0.01±0.01	0.09±0.14	0.19±0.02
	503_D	0.01±0.01	0.01±0.01	0.16±0.18	--	0.17±0.02	0.15±0.01	0.67±0.02	0.66±0.03
h_δ^2=0.15	1K (503_A+503_D)	0.01±0.01	0.01±0.01	0.12±0.15	--	0.16±0.02	0.14±0.02	0.56±0.03	0.57±0.02
σ_α^2=0.00	3K	0.01±0.02	0.01±0.02	0.14±0.17	--	0.04±0.03	0.03±0.02	0.20±0.12	0.32±0.02
	7K	0.01±0.02	0.01±0.01	0.13±0.15	--	0.06±0.05	0.05±0.04	0.24±0.09	0.37±0.02
σ_δ^2=0.18	40K	0.01±0.02	0.01±0.02	0.10±0.16	--	0.13±0.11	0.11±0.09	0.32±0.17	0.39±0.02
	41K (40K + 1K)	0.01±0.02	0.01±0.02	0.10±0.16	--	0.17±0.11	0.14±0.09	0.38±0.13	0.41±0.02
h_α^2=0.00	503_A	0.01±0.01	0.00±0.01	0.14±0.13	--	0.02±0.02	0.01±0.01	0.22±0.15	0.27±0.02
	503_D	0.01±0.01	0.01±0.01	0.15±0.17	--	0.44±0.05	0.30±0.03	0.81±0.02	0.79±0.02
h_δ^2=0.30	1K (503_A+503_D)	0.01±0.01	0.01±0.01	0.16±0.15	--	0.43±0.04	0.30±0.02	0.73±0.02	0.71±0.02
σ_α^2=0.00	3K	0.01±0.02	0.01±0.01	0.12±0.14	--	0.06±0.04	0.04±0.03	0.26±0.11	0.44±0.02
	7K	0.01±0.02	0.01±0.01	0.08±0.12	--	0.12±0.05	0.08±0.04	0.32±0.08	0.51±0.02
σ_δ^2=0.43	40K	0.01±0.01	0.00±0.01	0.09±0.10	--	0.26±0.09	0.18±0.06	0.45±0.08	0.55±0.02
	41K (40K + 1K)	0.01±0.01	0.00±0.01	0.08±0.10	--	0.36±0.08	0.25±0.06	0.53±0.06	0.57±0.02

The marker set of 503_A consisted of 503 causal SNP markers with additive effects and these markers were inter-QTL markers in the 1K data set when dominance effects were the only effects. The marker set of 503_D consisted of 503 causal SNP markers with dominance effects and these markers were inter-QTL markers in the 1K data set when additive effects were the only effects. h_α^2 is true additive heritability, h_δ^2 is true dominance heritability, σ_α^2 is true additive variance, σ_δ^2 is true dominance variance, $\hat{h}_\alpha^2$ is estimated additive heritability, $\hat{h}_\delta^2$ is estimated dominance heritability, $\hat{\sigma}_\alpha^2$ is estimated additive variance, σ_δ^2 is estimated dominance variance. R_a is predicted accuracy of GBLUP of breeding values, $\hat{R}_a$ is observed accuracy of GBLUP of breeding values, R_d is predicted accuracy of GBLUP of dominance deviations, $\hat{R}_d$ is observed accuracy of GBLUP of dominance deviations, R_g is predicted accuracy of GBLUP of genotypic values, $\hat{R}_g$ is observed accuracy of GBLUP of genotypic values.
