# Supplementary material for: Mixed Model Methods for Genomic Prediction and Variance Component Estimation of Additive and Dominance Effects Using SNP Markers
Source: PLoS One. 2014 Jan 30;9(1):e87666. doi: 10.1371/journal.pone.0087666 (PMC3907568; doi:10.1371/journal.pone.0087666)
Supplement: Table S3 — GBLUP Accuracies for breeding values, dominance deviations and genotypic values (mean ± standard deviation, n = 10 repeats). (PDF) [file pone.0087666.s003.pdf]

Table S3 GBLUP Accuracies for breeding values, dominance deviations and genotypic values (mean ± standard deviation, n = 10 repeats)

h^2	SNP type and density	R_a	$\hat{R}_a$	R_d	$\hat{R}_d$	R_g	$\hat{R}_g$
h_α^2=0.05, h_δ^2=0.05	1K_QTL	0.46±0.04	0.50±0.04	0.29±0.10	0.36±0.03	0.46±0.03	0.47±0.03
	1K_SNP	0.41±0.05	0.37±0.03	0.10±0.14	0.13±0.02	0.41±0.05	0.33±0.03
	2K (1K_QTL+1K_SNP)	0.44±0.04	0.47±0.03	0.20±0.13	0.30±0.02	0.44±0.03	0.43±0.03
	3K	0.41±0.04	0.42±0.04	0.07±0.13	0.19±0.02	0.41±0.05	0.38±0.03
	7K	0.40±0.05	0.43±0.04	0.08±0.13	0.22±0.02	0.41±0.05	0.39±0.03
	40K	0.41±0.06	0.43±0.04	0.10±0.14	0.23±0.02	0.41±0.06	0.40±0.03
	41K (40K+1K_QTL)	0.41±0.06	0.44±0.04	0.12±0.14	0.24±0.02	0.41±0.06	0.40±0.03
h_α^2=0.05, h_δ^2=0.15	1K_QTL	0.52±0.03	0.52±0.01	0.55±0.06	0.56±0.03	0.60±0.05	0.60±0.02
	1K_SNP	0.41±0.15	0.39±0.01	0.15±0.12	0.23±0.03	0.40±0.14	0.37±0.03
	2K (1K_QTL+1K_SNP)	0.49±0.05	0.49±0.02	0.50±0.07	0.50±0.03	0.57±0.05	0.55±0.02
	3K	0.43±0.11	0.44±0.02	0.20±0.13	0.32±0.03	0.43±0.11	0.43±0.04
	7K	0.44±0.08	0.45±0.02	0.27±0.13	0.36±0.03	0.45±0.10	0.46±0.03
	40K	0.42±0.10	0.46±0.01	0.35±0.14	0.39±0.03	0.48±0.11	0.48±0.03
	41K (40K+1K_QTL)	0.42±0.09	0.46±0.01	0.40±0.13	0.41±0.03	0.51±0.10	0.49±0.03
h_α^2=0.05, h_δ^2=0.30	1K_QTL	0.52±0.05	0.52±0.04	0.72±0.02	0.72±0.02	0.73±0.02	0.73±0.01
	1K_SNP	0.43±0.08	0.39±0.04	0.23±0.11	0.33±0.01	0.42±0.07	0.42±0.03
	2K (1K_QTL+1K_SNP)	0.48±0.04	0.48±0.04	0.66±0.03	0.66±0.02	0.69±0.02	0.68±0.01
	3K	0.44±0.04	0.43±0.04	0.27±0.11	0.45±0.02	0.45±0.03	0.51±0.04
	7K	0.44±0.04	0.44±0.04	0.32±0.11	0.52±0.02	0.48±0.02	0.56±0.04
	40K	0.42±0.05	0.44±0.05	0.47±0.07	0.56±0.02	0.55±0.05	0.61±0.01
	41K (40K+1K_QTL)	0.42±0.05	0.45±0.05	0.54±0.06	0.57±0.02	0.61±0.05	0.62±0.01
h_α^2=0.15, h_δ^2=0.05	1K_QTL	0.64±0.03	0.68±0.02	0.31±0.13	0.34±0.04	0.63±0.02	0.65±0.02
	1K_SNP	0.55±0.04	0.53±0.02	0.16±0.13	0.13±0.02	0.54±0.04	0.51±0.02
	2K (1K_QTL+1K_SNP)	0.60±0.03	0.65±0.02	0.29±0.11	0.29±0.03	0.60±0.02	0.62±0.02
	3K	0.56±0.03	0.59±0.02	0.16±0.13	0.18±0.02	0.56±0.03	0.56±0.02
	7K	0.58±0.02	0.59±0.02	0.12±0.10	0.21±0.02	0.57±0.02	0.57±0.02
	40K	0.57±0.02	0.60±0.02	0.17±0.12	0.23±0.02	0.58±0.02	0.58±0.02
	41K (40K+1K_QTL)	0.58±0.02	0.61±0.02	0.19±0.13	0.23±0.03	0.58±0.02	0.58±0.02
h_α^2=0.15,	1K_QTL	0.66±0.02	0.68±0.02	0.60±0.04	0.58±0.02	0.70±0.02	0.69±0.01
	1K_SNP	0.57±0.05	0.53±0.02	0.13±0.13	0.23±0.02	0.56±0.05	0.51±0.02
	2K (1K_QTL+1K_SNP)	0.62±0.03	0.65±0.02	0.52±0.05	0.51±0.02	0.66±0.03	0.66±0.01

h_δ^2=0.15	3K	0.58±0.04	0.58±0.02	0.11±0.12	0.33±0.02	0.57±0.04	0.56±0.02
	7K	0.58±0.04	0.59±0.02	0.23±0.14	0.38±0.02	0.59±0.04	0.59±0.02
	40K	0.59±0.04	0.60±0.02	0.33±0.12	0.41±0.02	0.62±0.04	0.61±0.02
	41K (40K+1K_QTL)	0.60±0.04	0.60±0.02	0.40±0.10	0.43±0.02	0.64±0.04	0.62±0.01
h_α^2 =0.15, h_δ^2=0.30	1K_QTL	0.66±0.03	0.70±0.02	0.74±0.02	0.73±0.01	0.77±0.02	0.78±0.01
	1K_SNP	0.58±0.03	0.54±0.02	0.16±0.12	0.32±0.02	0.57±0.04	0.52±0.03
	2K (1K_QTL+1K_SNP)	0.63±0.03	0.66±0.02	0.67±0.03	0.67±0.01	0.74±0.02	0.75±0.01
	3K	0.58±0.03	0.59±0.02	0.21±0.15	0.46±0.01	0.57±0.03	0.60±0.04
	7K	0.58±0.02	0.60±0.01	0.33±0.11	0.53±0.01	0.60±0.02	0.66±0.04
	40K	0.58±0.03	0.61±0.02	0.46±0.08	0.57±0.01	0.65±0.05	0.69±0.02
	41K (40K+1K_QTL)	0.59±0.03	0.61±0.02	0.55±0.06	0.58±0.01	0.69±0.05	0.70±0.01
h_α^2 =0.30, h_δ^2=0.05	1K_QTL	0.75±0.01	0.78±0.01	0.35±0.14	0.39±0.04	0.74±0.01	0.77±0.01
	1K_SNP	0.67±0.02	0.63±0.01	0.15±0.15	0.13±0.03	0.66±0.02	0.63±0.01
	2K (1K_QTL+1K_SNP)	0.72±0.02	0.75±0.01	0.30±0.17	0.32±0.03	0.72±0.01	0.74±0.01
	3K	0.66±0.02	0.68±0.01	0.10±0.12	0.19±0.02	0.66±0.02	0.68±0.01
	7K	0.66±0.01	0.70±0.02	0.12±0.13	0.23±0.02	0.66±0.01	0.70±0.01
	40K	0.67±0.01	0.70±0.01	0.23±0.13	0.25±0.02	0.68±0.02	0.70±0.02
	41K (40K+1K_QTL)	0.68±0.01	0.71±0.01	0.26±0.13	0.26±0.02	0.69±0.02	0.71±0.02
h_α^2 =0.30, h_δ^2=0.15	1K_QTL	0.77±0.01	0.79±0.01	0.61±0.02	0.61±0.02	0.78±0.01	0.79±0.00
	1K_SNP	0.68±0.01	0.63±0.01	0.12±0.14	0.23±0.02	0.67±0.01	0.62±0.01
	2K (1K_QTL+1K_SNP)	0.74±0.01	0.76±0.01	0.53±0.04	0.53±0.02	0.76±0.02	0.77±0.01
	3K	0.68±0.01	0.69±0.01	0.17±0.13	0.34±0.02	0.68±0.02	0.69±0.01
	7K	0.69±0.01	0.70±0.01	0.28±0.14	0.39±0.02	0.70±0.02	0.72±0.01
	40K	0.70±0.01	0.71±0.01	0.40±0.10	0.43±0.02	0.73±0.03	0.73±0.01
	41K (40K+1K_QTL)	0.70±0.01	0.71±0.01	0.46±0.08	0.44±0.02	0.75±0.03	0.74±0.01
h_α^2 =0.30, h_δ^2=0.30	1K_QTL	0.78±0.01	0.80±0.01	0.76±0.02	0.76±0.01	0.84±0.01	0.85±0.00
	1K_SNP	0.68±0.02	0.63±0.01	0.22±0.12	0.33±0.01	0.67±0.02	0.63±0.02
	2K (1K_QTL+1K_SNP)	0.74±0.01	0.76±0.01	0.70±0.02	0.69±0.01	0.82±0.01	0.82±0.00
	3K	0.67±0.02	0.68±0.01	0.28±0.05	0.47±0.01	0.67±0.02	0.71±0.01
	7K	0.69±0.02	0.70±0.01	0.39±0.07	0.55±0.01	0.70±0.03	0.76±0.02
	40K	0.69±0.02	0.71±0.01	0.52±0.05	0.59±0.01	0.76±0.02	0.80±0.01
	41K (40K+1K_QTL)	0.69±0.02	0.71±0.01	0.60±0.04	0.61±0.01	0.80±0.03	0.80±0.00

R_a is predicted accuracy of GBLUP of breeding values, $\hat{R}_a$ is observed accuracy of GBLUP of breeding values, R_d is predicted accuracy of GBLUP of dominance deviations, $\hat{R}_d$ is observed accuracy of GBLUP of dominance deviations, R_g is predicted accuracy of GBLUP of genotypic values, $\hat{R}_g$ is observed accuracy of GBLUP of genotypic values.
