# Supplementary material for: Mixed Model Methods for Genomic Prediction and Variance Component Estimation of Additive and Dominance Effects Using SNP Markers
Source: PLoS One. 2014 Jan 30;9(1):e87666. doi: 10.1371/journal.pone.0087666 (PMC3907568; doi:10.1371/journal.pone.0087666)
Supplement: Table S4 — GREML estimates of variance components and GBLUP accuracies with and without genomic relationships for phenotypes with additive and dominance effects of 1006 QTL (mean ± standard deviation, n = 10 repeats). (PDF) [file pone.0087666.s004.pdf]

Table S4 GREML estimates of variance components and GBLUP accuracies with and without genomic relationships for phenotypes with additive and dominance effects of 1006 QTL (mean ± standard deviation, *n*=10 repeats)

h^2 and σ^2	Definition		$\hat{\sigma}^2_\alpha$	$\hat{h}^2_\alpha$	R_a	$\hat{R}_a$	$\hat{\sigma}^2_\delta$	$\hat{h}^2_\delta$	R_d	$\hat{R}_d$
h^2_α=0.05	None	Mean	1.68×10^{-4}	1.67×10^{-4}	0.46	0.50	2.47×10^{-4}	2.47×10^{-4}	0.29	0.36
h^2_δ=0.05		SD	4.12×10^{-5}	4.19×10^{-5}	0.04	0.04	1.38×10^{-4}	1.41×10^{-4}	0.10	0.03
σ^2_α=0.06	I	Mean	0.06	0.06	0.46	0.50	0.04	0.03	0.29	0.36
σ^2_δ=0.06		SD	0.02	0.01	0.04	0.04	0.02	0.02	0.10	0.03
	II	Mean	0.06	0.06	0.46	0.50	0.04	0.03	0.29	0.36
		SD	0.02	0.01	0.04	0.04	0.02	0.02	0.10	0.03
	III	Mean	0.06	0.06	0.46	0.50	0.04	0.03	0.29	0.36
		SD	0.01	0.01	0.04	0.04	0.02	0.02	0.11	0.03
h^2_α=0.15	None	Mean	6.53×10^{-4}	6.79×10^{-4}	0.66	0.68	1.51×10^{-3}	1.57×10^{-3}	0.60	0.58
h^2_δ=0.15		SD	1.01×10^{-4}	1.26×10^{-4}	0.02	0.02	2.82×10^{-4}	3.36×10^{-4}	0.04	0.02
σ^2_α=0.21	I	Mean	0.24	0.17	0.66	0.68	0.23	0.16	0.60	0.58
σ^2_δ=0.21		SD	0.04	0.02	0.02	0.02	0.04	0.03	0.04	0.02
	II	Mean	0.24	0.17	0.66	0.68	0.22	0.16	0.60	0.58
		SD	0.04	0.02	0.02	0.02	0.04	0.03	0.04	0.02
	III	Mean	0.24	0.17	0.66	0.68	0.22	0.16	0.60	0.58
		SD	0.04	0.02	0.03	0.02	0.04	0.03	0.04	0.02
h^2_α=0.30	None	Mean	1.90×10^{-3}	1.91×10^{-3}	0.78	0.80	5.02×10^{-3}	5.06×10^{-3}	0.76	0.76
h^2_δ=0.30		SD	1.53×10^{-4}	1.93×10^{-4}	0.01	0.01	5.04×10^{-4}	6.63×10^{-4}	0.02	0.01
σ^2_α=0.75	I	Mean	0.71	0.29	0.78	0.80	0.75	0.31	0.76	0.76
σ^2_δ=0.75		SD	0.06	0.02	0.01	0.01	0.08	0.03	0.02	0.01
	II	Mean	0.69	0.29	0.78	0.80	0.74	0.31	0.76	0.76
		SD	0.06	0.02	0.01	0.01	0.07	0.03	0.02	0.01
	III	Mean	0.69	0.29	0.78	0.80	0.74	0.31	0.77	0.75
		SD	0.06	0.02	0.01	0.01	0.07	0.03	0.02	0.01

h^2_α is true additive heritability, h^2_δ is true dominance heritability, σ^2_α is true additive variance, σ^2_δ is true dominance variance, R_a is predicted accuracy of GBLUP of breeding values, $\hat{R}_a$ is observed accuracy of GBLUP of breeding values, R_d is predicted accuracy of GBLUP of dominance deviations, $\hat{R}_d$ is observed accuracy of GBLUP of dominance deviations, R_g is predicted accuracy of GBLUP

of genotypic values, $\hat{R}_g$ is observed accuracy of GBLUP of genotypic values, ‘None’: using the original mixed model of Equation 3 without genomic relationships.
