# Supplementary material for: Mixed Model Methods for Genomic Prediction and Variance Component Estimation of Additive and Dominance Effects Using SNP Markers
Source: PLoS One. 2014 Jan 30;9(1):e87666. doi: 10.1371/journal.pone.0087666 (PMC3907568; doi:10.1371/journal.pone.0087666)
Supplement: Table S5 — GBLUP accuracies in simulation data assuming random additive effects and directional dominance effects values (mean ± standard deviation, n = 10 repeats). (PDF) [file pone.0087666.s005.pdf]

Table S5 GBLUP accuracies in simulation data assuming random additive effects and directional dominance effects values (mean ± standard deviation, n = 10 repeats)

h^2	SNP set	R_a	$\hat{R}_a$	R_d	$\hat{R}_d$	R_g	$\hat{R}_g$
h_α^2=0.05 h_δ^2=0.05	1K_QTL	0.45±0.10[b]	0.40±0.06	0.43±0.06	0.50±0.04	0.50±0.05	0.51±0.03
	3K	0.38±0.07	0.29±0.06	0.32±0.07	0.36±0.03	0.41±0.07	0.43±0.03
	7K	0.37±0.09	0.31±0.07	0.37±0.05	0.38±0.03	0.44±0.06	0.44±0.03
	41K	0.36±0.12	0.32±0.07	0.40±0.07	0.38±0.03	0.48±0.06	0.43±0.03
h_α^2=0.05 h_δ^2=0.15	1K_QTL	0.48±0.02	0.40±0.03	0.61±0.03	0.67±0.02	0.63±0.03	0.67±0.01
	3K	0.45±0.06	0.27±0.04	0.48±0.05	0.50±0.02	0.55±0.04	0.55±0.02
	7K	0.45±0.06	0.30±0.04	0.52±0.04	0.52±0.01	0.58±0.03	0.56±0.01
	41K	0.42±0.08	0.31±0.04	0.62±0.04	0.50±0.02	0.67±0.03	0.55±0.02
h_α^2=0.05 h_δ^2=0.30	1K_QTL	0.48±0.05	0.41±0.05	0.72±0.01	0.79±0.02	0.73±0.01	0.78±0.01
	3K	0.48±0.05	0.25±0.04	0.57±0.03	0.63±0.02	0.62±0.03	0.67±0.01
	7K	0.46±0.06	0.29±0.04	0.61±0.02	0.65±0.02	0.66±0.03	0.68±0.02
	41K	0.38±0.10	0.32±0.04	0.72±0.03	0.64±0.02	0.76±0.02	0.67±0.02
h_α^2=0.15 h_δ^2=0.05	1K_QTL	0.65±0.04	0.60±0.02	0.42±0.07	0.45±0.03	0.64±0.04	0.62±0.02
	3K	0.56±0.04	0.46±0.04	0.29±0.10	0.31±0.02	0.55±0.04	0.52±0.02
	7K	0.57±0.04	0.48±0.04	0.30±0.12	0.32±0.02	0.58±0.04	0.53±0.02
	41K	0.56±0.05	0.50±0.04	0.41±0.09	0.31±0.03	0.61±0.06	0.54±0.02
h_α^2=0.15 h_δ^2=0.15	1K_QTL	0.66±0.04	0.61±0.02	0.58±0.03	0.65±0.03	0.69±0.03	0.71±0.01
	3K	0.59±0.02	0.46±0.03	0.43±0.08	0.48±0.02	0.61±0.03	0.61±0.01
	7K	0.60±0.02	0.49±0.03	0.46±0.09	0.50±0.02	0.65±0.03	0.63±0.01
	41K	0.59±0.02	0.51±0.02	0.55±0.06	0.49±0.02	0.70±0.03	0.62±0.02
h_α^2=0.15 h_δ^2=0.30	1K_QTL	0.66±0.03	0.62±0.03	0.72±0.02	0.78±0.02	0.76±0.02	0.80±0.01
	3K	0.60±0.03	0.43±0.03	0.54±0.04	0.62±0.01	0.66±0.03	0.71±0.01
	7K	0.59±0.04	0.46±0.03	0.59±0.04	0.64±0.02	0.71±0.03	0.72±0.01
	41K	0.57±0.04	0.50±0.04	0.71±0.03	0.62±0.02	0.81±0.04	0.71±0.01
h_α^2=0.30 h_δ^2=0.05	1K_QTL	0.77±0.02	0.75±0.01	0.44±0.06	0.48±0.04	0.76±0.02	0.76±0.02
	3K	0.66±0.03	0.61±0.02	0.24±0.12	0.31±0.04	0.66±0.03	0.65±0.02
	7K	0.67±0.03	0.64±0.02	0.28±0.14	0.31±0.04	0.68±0.03	0.67±0.02
	41K	0.68±0.03	0.66±0.02	0.39±0.10	0.32±0.04	0.72±0.02	0.68±0.02
h_α^2=0.30 h_δ^2=0.15	1K_QTL	0.78±0.02	0.75±0.01	0.62±0.03	0.67±0.02	0.79±0.01	0.79±0.01
	3K	0.68±0.03	0.59±0.01	0.39±0.09	0.49±0.02	0.69±0.03	0.70±0.02
	7K	0.69±0.03	0.62±0.01	0.44±0.08	0.50±0.03	0.72±0.04	0.72±0.01
	41K	0.70±0.03	0.64±0.02	0.53±0.07	0.51±0.02	0.78±0.04	0.72±0.01
h_α^2=0.30 h_δ^2=0.30	1K_QTL	0.80±0.01	0.77±0.01	0.74±0.02	0.80±0.01	0.80±0.01	0.85±0.01
	3K	0.69±0.02	0.58±0.01	0.54±0.03	0.61±0.01	0.73±0.02	0.78±0.01
	7K	0.70±0.02	0.62±0.01	0.57±0.03	0.63±0.01	0.78±0.01	0.80±0.01
	41K	0.71±0.02	0.65±0.01	0.69±0.03	0.63±0.02	0.87±0.03	0.80±0.01

h_α^2 is true additive heritability, h_δ^2 is true dominance heritability, R_a is predicted accuracy of GBLUP of breeding values, $\hat{R}_a$ is observed accuracy of GBLUP of breeding values, R_d is predicted accuracy of GBLUP of dominance deviations, $\hat{R}_d$ is observed accuracy of GBLUP of dominance deviations, R_g is predicted accuracy of GBLUP of genotypic values, $\hat{R}_g$ is observed accuracy of GBLUP of genotypic values.
