# Supplementary material for: Mixed Model Methods for Genomic Prediction and Variance Component Estimation of Additive and Dominance Effects Using SNP Markers
Source: PLoS One. 2014 Jan 30;9(1):e87666. doi: 10.1371/journal.pone.0087666 (PMC3907568; doi:10.1371/journal.pone.0087666)
Supplement: Text S1 — Proofs, formulations and simulation study. Part A: Derivations for the traditional quantitative genetics model of SNP markers with unequal and equal allele frequencies. Part B: Genomic prediction of additive and dominance effects for individuals without phenotypic observations. Part C: AI-REML implementation. Part D: Simulation study to evaluate GREML and GBLUP accuracies. (PDF) [file pone.0087666.s006.pdf]

Mixed model methods for genomic prediction and variance component estimation of additive and dominance effects using SNP markers

Yang Da*, Chunkao Wang, Shengwen Wang, Guo Hu
Department of Animal Science, University of Minnesota, Saint Paul, MN 55108, USA
*Corresponding Address: Yang Da, Email: yda@umn.edu

Text S1: Proofs, formulations and simulation study

Part A:
Derivations for the traditional quantitative genetics model of SNP markers with unequal and equal allele frequencies

The two alternative alleles of the SNP are denoted by A_1 and A_2 with allele frequencies $p(A_1) = p$ and $p(A_2) = q$. Hardy-Weinberg equilibrium (HWE) is assumed so that the genotypic array satisfies: $p^2A_1A_1 + 2pqA_1A_2 + q^2A_2A_2 = (pA_1 + qA_2)^2$. Details for deriving the traditional quantitative genetics model of additive and dominance effects are summarized in Table A1.1, which is essentially the same as in Falconer and Mackay [1] except that the genotypic value for each SNP genotype used a general notation g_{ij} (i,j = 1,2) to allow the derivation of the relationship between the genotypic values and additive and dominance effects.

Table A1.1 Calculation of population mean and average effect ($N = N_{11} + N_{12} + N_{22}$)

Genotype	A_1A_1	A_1A_2	A_2A_2
Number of individual	N_{11}	N_{12}	N_{22}
Genotypic frequency: general expression	$P_{11} = N_{11}/N$	$P_{12} = N_{12}/N$	$P_{22} = N_{22}/N$
Genotypic frequency: under HWE	p^2	$2pq$	q^2
Number of A_1	2	1	0
Number of A_2	0	1	2
Genotypic value	g_{11}	g_{12}	g_{22}

Let μ = the common mean of all genotypic values = $p^2g_{11} + 2pqg_{12} + q^2g_{22}$, μ_i = the average genotypic value for all genotypes carrying A_i allele, i =1,2, with $\mu_1 = pg_{11} + qg_{12}$ and $\mu_2 = pg_{12} + qg_{22}$. Then,

a_1 = average effect of A_1 allele = $\mu_1 - \mu = q\alpha$
a_2 = average effect of A_2 allele = $\mu_2 - \mu = -p\alpha$

where

α = the average effect of gene substitution
$= a_1 - a_2 = \mu_1 - \mu_2 = p(g_{11}) + (q - p)g_{12} - qg_{22}$.

The partition of a genotypic value into breeding value and dominance deviation based on Table A1.1 has the same results as in [1] and are summarized in Table A1.2.

Table A1.2 Breeding value and dominance deviation

Genotype	A_1A_1	A_1A_2	A_2A_2
Corrected genotypic value	$t_{11} = g_{11} - \mu$	$t_{12} = g_{12} - \mu$	$t_{22} = g_{22} - \mu$
Breeding value	$a_{11} = 2a_1 = 2q\alpha$	$a_{12} = a_1 + a_2 = (q-p)\alpha$	$a_{22} = 2a_2 = -2p\alpha$
Dominance deviation	$d_{11} = t_{11} - a_{11} = -2q^2\delta$	$d_{12} = t_{12} - a_{12} = 2pq\delta$	$d_{22} = t_{22} - a_{22} = -2p^2\delta$

In Table A1.2,

$$\alpha = \frac{1}{2}(a_{11} - a_{22}) = a_1 - a_2 \text{ = the average effect of gene substitution;}$$

$$\delta = d_{12} - \frac{1}{2}(d_{11} + d_{22}) = g_{12} - \frac{1}{2}(g_{11} + g_{22}) \text{ = dominance effect.}$$

Based on the above results, the traditional quantitative genetics model that partitions a genotypic value into additive and dominance effects is:

$$g_{ij} = \text{mean} + (\text{breeding value}) + (\text{dominance deviation}) = \mu + a_{ij} + d_{ij} \quad i,j = 1,2; \text{ or,}$$

$$g_{11} = \mu + (2q\alpha) + (-2q^2\delta)$$
$$g_{12} = \mu + [(q - p)\alpha] + (2pq\delta)$$
$$g_{22} = \mu + (-2p\alpha) + (-2p^2\delta)$$

In matrix notations, the above equations lead to Equation 2 in the main text, i.e.,

$$\begin{pmatrix} g_{11} \\ g_{12} \\ g_{22} \end{pmatrix} = \begin{pmatrix} 1 & 2q & -2q^2 \\ 1 & q-p & 2pq \\ 1 & -2p & -2p^2 \end{pmatrix} \begin{pmatrix} \mu \\ \alpha \\ \delta \end{pmatrix} \tag{2}$$

Assuming equal allele frequencies, $p = q = ½$, and reparameterizing μ as $\mu^* = \mu - ½\delta$, Equation 2 reduces to:

$$\begin{pmatrix} g_{11} \\ g_{12} \\ g_{22} \end{pmatrix} = \begin{pmatrix} 1 & 1 & 0 \\ 1 & 0 & 1 \\ 1 & -1 & 0 \end{pmatrix} \begin{pmatrix} \mu^* \\ \alpha \\ \delta \end{pmatrix} \tag{A.1}$$

If μ^* is further reparameterized as $\mu^{**} = \mu^* - \alpha = \mu - \frac{1}{2}\delta - \alpha$, equation (A.1) becomes:

$$\begin{pmatrix} g_{11} \\ g_{12} \\ g_{22} \end{pmatrix} = \begin{pmatrix} 1 & 2 & 0 \\ 1 & 1 & 1 \\ 1 & 0 & 0 \end{pmatrix} \begin{pmatrix} \mu^{**} \\ \alpha \\ \delta \end{pmatrix} \qquad \text{(A.2)}$$

Equation A.1 is the basis for the SNP coding of (-1)-0-1 for additive effect and 0-1-0 coding for dominance effect, and Equation A.2 is the basis for the SNP coding of 0-1-2 for additive effect and 0-1-0 coding for dominance effect. These two models are the reparameterized models of the original quantitative genetics model of Equation 2 under the assumption of equal allele frequencies. With unequal allele frequencies, the additive effects in the equal-frequency models of Equations A.1 and A.2 are not 'breeding values' defined in the traditional quantitative genetics model of Equation 2.

Part B:
Genomic prediction of additive and dominance effects for individuals without phenotypic observations

For m>q, where the additive and dominance relationship matrices are invertible, two methods of Henderson [2] for animals without records can be used for calculating GBLUP of individuals without phenotypic observations. The first method is to augment the MME for individuals with phenotypic observation by adding individuals without phenotypic observations to GBLUP-CE of Equations 12-13, adding a column vector of 0's to the **y** vector, and adding a submatrix of 0's to each of the **X** and **Z** matrices corresponding to individuals without phenotypic observations. The advantage of this approach is that no new formulations are required for GBLUP and reliability of individuals without phenotypic observations because GBLUP and reliability can be calculated from the same GBLUP-CE. The disadvantage is the increased number of equations in GBLUP-CE for simultaneous solutions.

The second method for m>q is to predict animals without observations based on animals with observations. With the addition of SNP markers for individuals without phenotypic records, the $\mathbf{A}_g$ and $\mathbf{D}_g$ matrices of Equations 6-7 can be partitioned as:

$$\mathbf{A}_g = \begin{pmatrix} \mathbf{A}_{11} & \mathbf{A}_{10} \\ \mathbf{A}_{01} & \mathbf{A}_{00} \end{pmatrix}, \mathbf{D}_g = \begin{pmatrix} \mathbf{D}_{11} & \mathbf{D}_{10} \\ \mathbf{D}_{01} & \mathbf{D}_{00} \end{pmatrix}$$

where $\mathbf{A}_{11}$ = q×q matrix of additive correlations among individuals with phenotypic records, $\mathbf{A}_{10}$ = q×q_0 matrix of additive correlations between individuals with phenotypic observations and individuals without phenotypic observations, $\mathbf{A}_{00}$ = q_0×q_0 matrix of additive correlations among individuals without phenotypic observations, $\mathbf{D}_{11}$ = q×q matrix of dominance correlations among individuals with phenotypic observations, $\mathbf{D}_{10}$ = q×q_0 matrix of dominance correlations between individuals with and without phenotypic observations, $\mathbf{D}_{00}$ = q_0×q_0 matrix of dominance correlations among individuals without phenotypic observations, and where q_0 = number of animals without observations. Then, the GBLUP-CE of individuals without phenotypic observations can be obtained as:

$$\hat{\mathbf{a}}_0 = \mathbf{A}_{01}\mathbf{A}_{11}^{-1}\hat{\mathbf{a}} = \sigma_\alpha^2\mathbf{A}_{01}\mathbf{Z}\mathbf{V}^{-1}(\mathbf{y} - \mathbf{X}\hat{\mathbf{b}}) = \sigma_\alpha^2\mathbf{A}_{01}\mathbf{Z}\mathbf{P}\mathbf{y} \quad \text{(B.1)}$$

$$\hat{\mathbf{d}}_0 = \mathbf{D}_{01}\mathbf{D}_{11}^{-1}\hat{\mathbf{d}} = \sigma_\delta^2\mathbf{D}_{01}\mathbf{Z}\mathbf{V}^{-1}(\mathbf{y} - \mathbf{X}\hat{\mathbf{b}}) = \sigma_\delta^2\mathbf{D}_{01}\mathbf{Z}\mathbf{P}\mathbf{y} \quad \text{(B.2)}$$

$$\hat{\mathbf{g}}_0 = \hat{\mathbf{a}}_0 + \hat{\mathbf{d}}_0 \quad \text{(B.3)}$$

where $\hat{\mathbf{a}}_0$ = genomic estimated breeding value vector for individuals without phenotypic observations, $\hat{\mathbf{d}}_0$ = genomic estimated dominance deviation vector for individuals without phenotypic observations, $\hat{\mathbf{a}}$ and $\hat{\mathbf{d}}$ are from GBLUP-CE of Equations 12-13, and **P** is defined by Equation 14. Reliabilities of Equations B.1-B.3 are:

$$\mathrm{R}^2_{ai} = \sigma^2_\alpha\left(\mathbf{A}_{01}\mathbf{Z'PZA}_{10}\right)_{ii} / a_{ii}$$
$$\mathrm{R}^2_{di} = \sigma^2_\delta\left(\mathbf{D}_{01}\mathbf{Z'PZD}_{10}\right)_{ii} / d_{ii}$$
$$\mathrm{R}^2_{gi} = \frac{\left(\sigma^4_\alpha\mathbf{A}_{01}\mathbf{Z'PZA}_{10} + \sigma^2_\alpha\mathbf{A}_{01}\mathbf{Z'PZD}_{10}\sigma^2_\delta + \sigma^2_\delta\mathbf{D}_{01}\mathbf{Z'PZA}_{10}\sigma^2_\alpha + \sigma^4_\delta\mathbf{D}_{01}\mathbf{Z'PZD}_{10}\right)_{ii}}{a_{ii}\sigma^2_\alpha + d_{ii}\sigma^2_\delta}$$

For $q>m$, GBLUP-QM of individuals without phenotypic observations can be obtained as:

$$\hat{\mathbf{a}}_0 = \mathbf{T}_{\alpha 0}\hat{\boldsymbol{\alpha}} = \sigma^2_\alpha\mathbf{A}_{01}\mathbf{Z'Py} \quad \text{(B.4)}$$
$$\hat{\mathbf{d}}_0 = \mathbf{T}_{\delta 0}\hat{\boldsymbol{\delta}} = \sigma^2_\delta\mathbf{D}_{01}\mathbf{Z'Py} \quad \text{(B.5)}$$
$$\hat{\mathbf{g}}_0 = \hat{\mathbf{a}}_0 + \hat{\mathbf{d}}_0 \quad \text{(B.6)}$$

where $\mathbf{T}_{\alpha 0}$ and $\mathbf{T}_{\delta 0}$ are given by Equations 4-5 but are calculated using SNP of individuals without phenotypic observations, and $\hat{\boldsymbol{\alpha}}$ and $\hat{\boldsymbol{\delta}}$ are from GBLUP-QM of Equation 19. Note that GBLUP-QM of Equations B.4-B.6 and GBLUP-CE of Equations B.1-B.3 are mathematically equivalent. Reliabilities of GBLUP from Equations B.4-B.6 are:

$$\mathrm{R}^2_{ai} = 1 - \lambda_{\boldsymbol{\alpha}}\left(\mathbf{T}_{\alpha 0}\mathbf{C}^{\alpha\alpha}\mathbf{T}_{\alpha 0}'\right)_{ii} / a_{ii}$$
$$\mathrm{R}^2_{di} = 1 - \lambda_\delta\left(\mathbf{T}_{\delta 0}\mathbf{C}^{\delta\delta}\mathbf{T}_{\delta 0}'\right)_{ii} / d_{ii}$$
$$\mathrm{R}^2_{gi} = 1 - \sigma^2_e\left(\mathbf{T}_{\alpha 0}\mathbf{C}^{\alpha\alpha}\mathbf{T}_{\alpha 0}' + \mathbf{T}_{\alpha 0}\mathbf{C}^{\alpha\delta}\mathbf{T}_{\delta 0}' + \mathbf{T}_{\delta 0}\mathbf{C}^{\delta\alpha}\mathbf{T}_{\alpha 0}' + \mathbf{T}_{\delta 0}\mathbf{C}^{\delta\delta}\mathbf{T}_{\delta 0}'\right)_{ii} / \left(a_{ii}\sigma^2_\alpha + d_{ii}\sigma^2_\delta\right)$$

Part C:
AI-REML implementation

The general formula of AI-REML [3-5] can be written as:

$$\boldsymbol{\Theta}^{(i+1)} = \boldsymbol{\Theta}^{(i)} + \left(\mathbf{AI}^{(i)}\right)^{-1}\boldsymbol{\Delta}^{(i)}$$

where $\boldsymbol{\Theta} = (\sigma^2_\alpha, \sigma^2_\delta, \sigma^2_e)'$, i = iteration number, **AI** = matrix of average information, and $\boldsymbol{\Delta} = (\Delta_\alpha, \Delta_\delta, \Delta_e)'$ = the partial derivatives of the log-likelihood function (L) with respect to each variance component. For GREML_CE, the **AI** and **Δ** can use the formulae in [3] except notation changes. For GREML_QM, **Δ** can also use the formulae in [5] except notation changes. The **AI** matrix for GREML_QM with the absorption of fixed effects can be written as:

$$\mathbf{AI} = \frac{1}{2}\begin{pmatrix} \hat{\boldsymbol{\alpha}}'\mathbf{B}_1\mathbf{Z}_1\hat{\boldsymbol{\alpha}}/(\sigma^2_\alpha)^3 & \hat{\boldsymbol{\alpha}}'\mathbf{B}_1\mathbf{Z}_2\hat{\boldsymbol{\delta}}/[(\sigma^2_\alpha)^2\sigma^2_\delta] & \hat{\boldsymbol{\alpha}}'\mathbf{B}_1\hat{\mathbf{e}}/[(\sigma^2_\alpha)^2\sigma^2_e] \\ \hat{\boldsymbol{\alpha}}'\mathbf{B}_1\mathbf{Z}_2\hat{\boldsymbol{\delta}}/[(\sigma^2_\alpha)^2\sigma^2_\delta] & \hat{\boldsymbol{\delta}}'\mathbf{B}_2\mathbf{Z}_2\hat{\boldsymbol{\delta}}/(\sigma^2_\delta)^3 & \hat{\boldsymbol{\delta}}'\mathbf{B}_2\hat{\mathbf{e}}/[(\sigma^2_\delta)^2\sigma^2_e] \\ \hat{\boldsymbol{\alpha}}'\mathbf{B}_1\hat{\mathbf{e}}/[(\sigma^2_\alpha)^2\sigma^2_e] & \hat{\boldsymbol{\delta}}'\mathbf{B}_2\hat{\mathbf{e}}/[(\sigma^2_\delta)^2\sigma^2_e] & \hat{\mathbf{e}}'\mathbf{B}_3\hat{\mathbf{e}}/(\sigma^2_e)^3 \end{pmatrix}$$

where

$$\mathbf{B}_1 = \mathbf{C}^{\alpha\alpha}\mathbf{Z}_1'\mathbf{M} + \mathbf{C}^{\alpha\delta}\mathbf{Z}_2'\mathbf{M} = \sigma^2_\alpha\mathbf{Z}_1'\mathbf{P} = \mathbf{Z}_1'\mathbf{B}_3/\sigma^2_e$$
$$\mathbf{B}_2 = \mathbf{C}^{\alpha\delta}\mathbf{Z}_1'\mathbf{M} + \mathbf{C}^{\delta\delta}\mathbf{Z}_2'\mathbf{M} = \sigma^2_\alpha\mathbf{Z}_2'\mathbf{P} = \mathbf{Z}_2'\mathbf{B}_3/\sigma^2_e$$
$$\mathbf{B}_3 = \mathbf{M} - \mathbf{M}\mathbf{Z}_u\mathbf{H}^{-1}\mathbf{Z}_u'\mathbf{M} = \sigma^2_e\mathbf{P}$$

and where $\mathbf{Z}_1 = \mathbf{Z}\mathbf{T}_\alpha$ and $\mathbf{Z}_2 = \mathbf{Z}\mathbf{T}_\delta$, $\mathbf{M} = \mathbf{I}_N - \mathbf{X}(\mathbf{X}'\mathbf{X})^-\mathbf{X}'$, $\mathbf{H}^{-1}$, $\mathbf{C}^{\alpha\alpha}$, $\mathbf{C}^{\alpha\delta}$, and $\mathbf{C}^{\delta\delta}$ are defined by Equation 22, **P** is defined by Equation 14, and $\mathbf{Z}_u = (\mathbf{Z}_1, \mathbf{Z}_2)$. In terms of computing implementation, the calculation of **M**, which is a large matrix not calculated for GREML, can be avoided, e.g., by calculating $\hat{\boldsymbol{\alpha}}'\mathbf{Z}_1'\mathbf{M}'$ as $\hat{\boldsymbol{\alpha}}'\mathbf{Z}_1' - \hat{\boldsymbol{\alpha}}'\mathbf{Z}_1'\mathbf{X}(\mathbf{X}'\mathbf{X})^-\mathbf{X}'$. Other terms in the **AI** matrix can be calculated in the same way to avoid the need to store **M**.

Part D:
Simulation study to evaluate GREML and GBLUP accuracies

Simulated values of quantitative trait loci (QTL) and phenotypes were generated to evaluate the performance of GBLUP and GREML for additive and dominance effects within the training data set. The central question to be answered by the simulation study was the accuracies of GREML and GBLUP for causal variants, SNP markers with various marker densities, and various heritability levels. For this purpose, individuals with SNP markers and known breeding values and dominance deviations (individuals in simulated training data set), rather than individuals in the validation data set, were the appropriate choice. The SNP data with 45,878 markers for 1654 Holstein cows [6,7] were used to base the data simulation on a real SNP structure. Minor allele frequency of 0.05 was required and 40,544 markers covering all 29 autosomes and the X chromosome satisfied this condition in the final SNP data set. The 40,544 markers were divided into 1000 segments in equal distance and 1006 bordering markers of the 1000 segments were selected as QTL with an average QTL spacing approximately 2.64 Mb. The QTL genotypic values were generated with a mixture of additive and dominance effects in the order of 'A-D-A-D-A-D...' for each chromosome, where 'A' and 'D' denote additive and dominance effects, respectively. True QTL values were generated using Equation 2 by setting $\mu = \delta = 0$ and using α values from a uniform [-1,1] distribution for generating breeding values of the markers, and by setting $\mu = \alpha = 0$ and using δ values from a uniform [-1,1] distribution for generating dominance deviations. The resulting breeding values and dominance deviations had normal distributions, see Figure below.

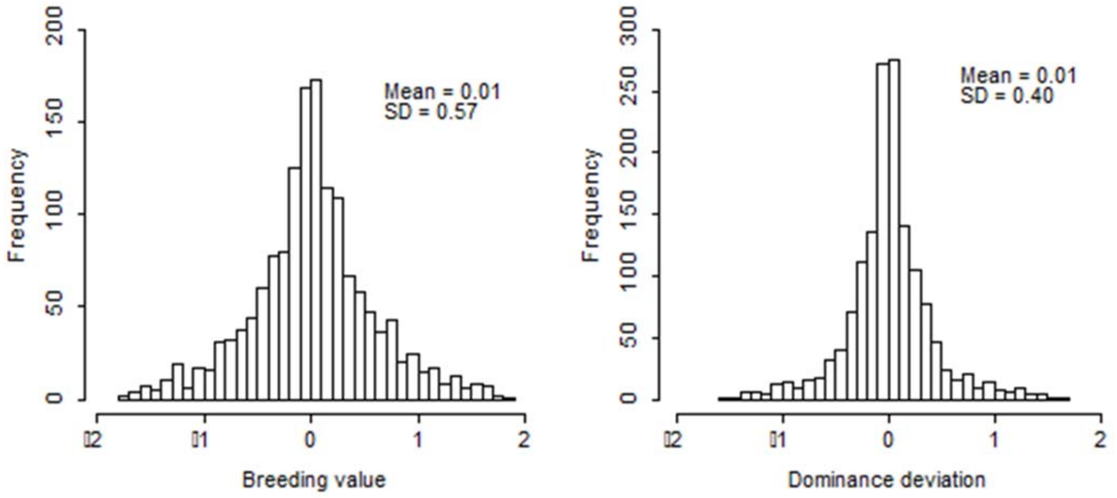

Figure D.1 Distribution of breeding values and dominance deviations assuming uniform distribution of α and δ values. Left: breeding values. Right**:** dominance deviations.

Random residuals were generated as a standardized normal variable, i.e., e~N(0,1). The total additive value and the total dominance value each was scaled to achieve a target heritability level. Let α_0 is the original additive value as the summation of the additive

values of all QTL, with variance of $Var(\alpha_0) = \sigma^2_{\alpha_0}$, α_1 is the transformed additive value with variance of $Var(\alpha_1) = \sigma^2_{\alpha_1}$ to achieve the target heritability of h^2_α, δ_0 is the original dominance value as the summation of the dominance values of all QTL with variance of $Var(\delta_0) = \sigma^2_{\delta_0}$, δ_1 is the transformed dominance value with variance of $Var(\delta_1) = \sigma^2_{\delta_1}$ to achieve the target heritability of h^2_δ, $\sigma^2_y = \sigma^2_\alpha + \sigma^2_\delta + \sigma^2_e$ (noting $\sigma^2_e = 1.00$) is the phenotypic variance after transforming the additive and dominance values to achieve the target additive and dominance heritabilities, $h^2_\alpha = \sigma^2_\alpha / \sigma^2_y$ is the target additive heritability, and $h^2_\delta = \sigma^2_\delta / \sigma^2_y$ is the target dominance heritability. Then, the transformations of additive and dominance values to achieve the target heritabilities are: $\alpha_1 = \beta_\alpha \alpha_0$, and $\delta_1 = \beta_\delta \delta_0$, where $\beta_\alpha = \sqrt{\frac{h^2_\alpha}{1 - h^2_\alpha - h^2_\delta} \sigma^2_{\alpha_0}}$ and $\beta_\delta = \sqrt{\frac{h^2_\delta}{1 - h^2_\alpha - h^2_\delta} \sigma^2_{\delta_0}}$. The phenotypic observations were generated as $y = QTL + e$ for true h^2_α and h^2_α levels of 0.05, 0.15 and 0.30. This range of true heritability levels translates into approximately 0.00005-0.0003 of the phenotypic variation for each of the 1006 QTL. These small SNP effects would be undetectable through genome-wide association analysis. Seven marker sets were studied for the following purposes: the 1K_QTL set for studying the accuracies of causal variants, the 1K_SNP set for studying the accuracies of inter-QTL markers that were approximately in equal numbers as in the 1K_QTL set, the 2K and 41K for studying the accuracy changes by adding inter-QTL markers with different densities to the causal variants, and the 3K, 7K and 40K inter-QTL markers for studying the accuracies of linked markers with different densities. These marker sets are summarized in the table below.

Table D.1. SNP marker sets used in the simulation study

SNP set	Number of SNP markers	Average spacing (kb)	Type of SNP markers
1K_QTL	1006	2641	Causal SNP
1K_SNP	975	2725	Inter-QTL SNP
2K	1981 (=1006 + 975)	1341	1K_QTL + 1K_SNP
3K	3000	886	Inter-QTL SNP
7K	7000	380	Inter-QTL SNP
40K	39,538	67	Inter-QTL SNP
41K	40,544	66	40K + 1K_QTL

GREML estimates of additive, dominance and residual variances, as well as additive and dominance heritabilities, were obtained using the GVCBLUP computer package that implemented methods in this study [8]. GBLUP of additive and dominance effects were obtained as the solutions at the last iteration of GREML. Accuracy of GREML for variance components and heritabilities was measured by bias, the mean square error (MSE), relative bias and relative MSE, where

$$\text{bias} = (\text{mean of estimates}) - (\text{true value})$$
$$\text{MSE} = (\text{bias})^2 + (\text{variance of estimates})$$
$$(\text{relative bias}) = \text{bias}/(\text{true value})$$
$$(\text{relative MSE}) = \sqrt{\text{MSE}}/(\text{true value}).$$

Each predicted GBLUP accuracy was calculated as the square root of the reliability measure developed in this study, and observed GBLUP accuracy was calculated as the correlation between the GBLUP and the true breeding values, dominance deviations or genetic values.

To investigate dominance GBLUP accuracy for phenotypes with heterosis, we generated simulation data set assuming positive dominance deviation for each heterozygous genotype and negative dominance deviation for each homozygous genotype by setting the 'δ' value to '1' in Equation 2. The resulting simulation data had a skewed distribution with mean of '-0.3' due to the fact that the Holstein population used in the simulation study had more homozygous genotypes than heterozygous genotypes, see figure below.

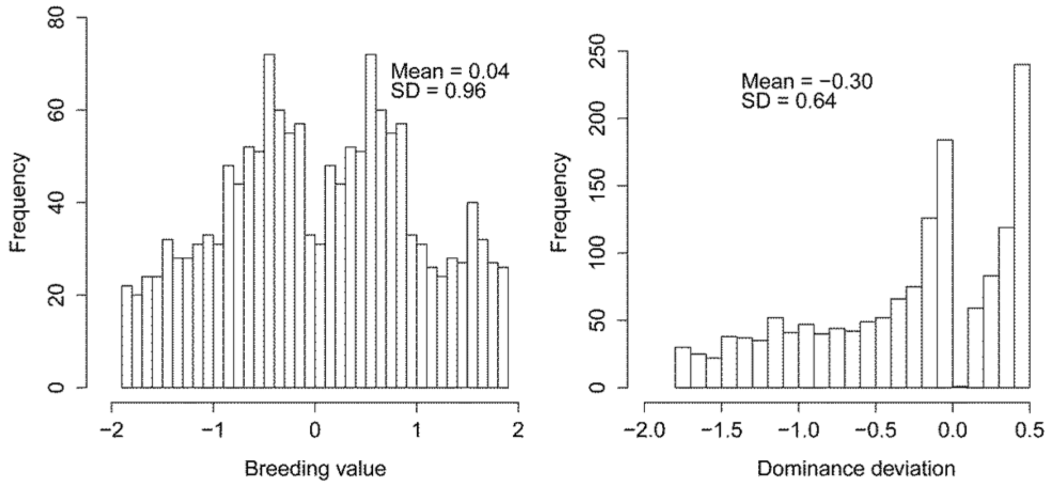

Figure D.2 Distribution of simulated data assuming random additive effects and directional dominance effects. Left: Distribution of breeding values. Right**:** Distribution of dominance deviations.

References

1. Falconer DS, Mackay TFC (1996) Introduction to Quantitative Genetics (4th edition). Harlow, Essex, UK: Longmans Green.
2. Henderson C (1977) Best linear unbiased prediction of breeding values not in the model for records. Journal of Dairy Science 60: 783-787.
3. Gilmour AR, Thompson R, Cullis BR (1995) Average information REML: an efficient algorithm for variance parameter estimation in linear mixed models. Biometrics: 1440-1450.
4. Johnson D, Thompson R (1995) Restricted maximum likelihood estimation of variance components for univariate animal models using sparse matrix techniques and average information. Journal of dairy science 78: 449-456.
5. Lee SH, van der Werf JH (2006) An efficient variance component approach implementing an average information REML suitable for combined LD and linkage mapping with a general complex pedigree. Genetics Selection Evolution 38: 1-19.
6. Cole JB, Wiggans GR, Ma L, Sonstegard TS, Lawlor TJ, et al. (2011) Genome-wide association analysis of thirty one production, health, reproduction and body conformation traits in contemporary US Holstein cows. BMC genomics 12: 408.
7. Ma L, Wiggans G, Wang S, Sonstegard T, Yang J, et al. (2012) Effect of sample stratification on dairy GWAS results. BMC genomics 13: 536.
8. Wang C, Prakapenka D, Wang S, Runesha HB, Da Y (2013) GVCBLUP: a computer package for genomic prediction and variance component estimation of additive and dominance effects using SNP markers. Version 3.7. Department of Animal Science, University of Minnesota. [http://animalgene.umn.edu]
